# Decoding Covert Speech From EEG-A Comprehensive Review

**DOI:** 10.3389/fnins.2021.642251

**Published:** 2021-04-29

**Authors:** Jerrin Thomas Panachakel, Angarai Ganesan Ramakrishnan

**Affiliations:** Medical Intelligence and Language Engineering Laboratory, Department of Electrical Engineering, Indian Institute of Science, Bangalore, India

**Keywords:** imagined speech, brain-computer interfaces (BCI), neurorehabilitation, electroencephalogram (EEG), speech imagery, covert speech, inner speech

## Abstract

Over the past decade, many researchers have come up with different implementations of systems for decoding covert or imagined speech from EEG (electroencephalogram). They differ from each other in several aspects, from data acquisition to machine learning algorithms, due to which, a comparison between different implementations is often difficult. This review article puts together all the relevant works published in the last decade on decoding imagined speech from EEG into a single framework. Every important aspect of designing such a system, such as selection of words to be imagined, number of electrodes to be recorded, temporal and spatial filtering, feature extraction and classifier are reviewed. This helps a researcher to compare the relative merits and demerits of the different approaches and choose the one that is most optimal. Speech being the most natural form of communication which human beings acquire even without formal education, imagined speech is an ideal choice of prompt for evoking brain activity patterns for a BCI (brain-computer interface) system, although the research on developing real-time (online) speech imagery based BCI systems is still in its infancy. Covert speech based BCI can help people with disabilities to improve their quality of life. It can also be used for covert communication in environments that do not support vocal communication. This paper also discusses some future directions, which will aid the deployment of speech imagery based BCI for practical applications, rather than only for laboratory experiments.

## 1. Introduction

We, as human beings, keep talking within us most of the times. We rehearse over and over again how to manage a particular difficult situation, what to talk to a prospective customer, how to answer certain critical questions in an interview, and so on. This speech, unlike the overt speech in a conversation with another person, is imagined and hence, there is no movement of the articulators. Thus, imagined speech is a very common, daily phenomenon with every human being. Even when someone's muscles are paralyzed and one is not able to move one's articulators, one can still imagine speaking or actively think.

Imagined speech, active thought or covert speech is defined as the voluntary imagination of speaking something without actually moving any of the articulators. The interest in decoding imagined speech dates back to the days of Hans Berger, the German neurologist who recorded the first human EEG in the year 1928. It is said that Hans Berger developed EEG as a tool for synthetic telepathy, which involves imagined speech (Keiper, 2006; Kaplan, 2011). In the year 1967, Dewan attempted transmitting letters as Morse code using EEG (Dewan, 1967). Speech being the natural form of communication for human beings, researchers across the globe are trying to develop BCI (brain-computer interface) systems based on speech imagery instead of motor imagery.

A BCI system translates the distinct electrical activities of the brain into commands for obtaining different desired results from an external device. BCI systems can aid patients who have lost the control over their voluntary muscles in their day-to-day activities, from controlling the lighting in a room to using a personal computer (Abdulkader et al., 2015). BCI systems make use of different electrophysiological and neuroimaging modalities like electroencephalogram (EEG), electrocorticogram (ECoG), fMRI (functional magnetic resonance imaging), fNIRS (functional near-infrared spectroscopy), and intracortical electroencephalography (ICE) for capturing the electrical activity of the brain. Refer Hiremath et al. (2015) for a review on BCI systems using ECoG and ICE. Currently available BCI systems using EEG depend on motor imagery (Onose et al., 2012; Kevric and Subasi, 2017), event-related potential (ERP) (Sellers et al., 2006; Mugler et al., 2010; Xu et al., 2018b; Fouad et al., 2020) or steady state visually evoked potentials (SSVEP) (Müller-Putz et al., 2005; Han et al., 2018; Ojha and Mukul, 2020) for generating consistent and reliable brain signals that can be accurately identified by the system. P300-speller based BCI system (Guan et al., 2004; Guy et al., 2018; Arvaneh et al., 2019; Lu et al., 2019; Al-Nuaimi et al., 2020) is a quite successful BCI system. Nevertheless, some of these BCI systems are either constrained by the limited number of distinct prompts possible and/or by the difficulty in training someone to use these systems. Using imagined speech for evoking the brain activity pattern has several advantages such as provision for larger number of prompts (which in turn leads to higher degrees of freedom) than what is possible with motor imagery. In addition to all the possible applications of a general BCI system based on motor imagery, a high-performance BCI system based on speech imagery, in conjunction with a text to speech (TTS) system, can be used by those with speech disabilities to communicate with others. It can also be used for covert communication in environments such as war fields, where overt vocal communication is difficult (Allison et al., 2007; Bogue, 2010).

This paper reviews the recent literature in the field of decoding imagined speech from EEG, mainly from the point of view of the considerations behind the choice of various parameters in designing and developing an effective system. EEG based systems have the following advantages compared to systems based on neuroimaging techniques such as fMRI, fNIRS, and ECoG due to the following reasons:

- EEG is cheaper and non-invasive (Kayagil et al., 2007; Zanzotto and Croce, 2010; Illman et al., 2020; Tait et al., 2020).
- EEG has good temporal resolution although ECoG has higher temporal resolution (Yi et al., 2013; Hecht and Stout, 2015; Ghafoor et al., 2019). However, studies have shown that volume conduction and increased distance between the cortical sources and electrodes limit the temporal resolution of EEG (Law et al., 1993; Burle et al., 2015).
- One issue with using EEG is that the setup time is very high, especially for high density EEG systems. This problem can be alleviated by identifying the EEG channels that significantly influence the performance of the system and creating custom EEG electrode caps with only these electrodes. The setup and preparation times can also be reduced by using dry electrodes instead of gel based electrodes (Sellers et al., 2009; Grozea et al., 2011; Guger et al., 2012).

Nevertheless, the following factors limit the application of EEG based BCI systems:

- EEG has lower signal-to-noise ratio (SNR) than the other modalities. It is almost always corrupted by artifacts such as muscular artifacts (Eberle et al., 2012; Liu, 2019).
- EEG has limited spectral and spatial resolution (Peled et al., 2001; Lakshmi et al., 2014).
- Recording EEG for longer duration is challenging since the conductive gel or the saline solution applied for reducing the electrode impedance dries up over time, thus increasing the electrode impedance (Guger et al., 2012; Xu et al., 2018a).
- A trained personnel is required for placing the EEG electrode cap.

Table 1 compares various electrophysiological and neuroimaging techniques used for decoding imagined speech from EEG.

**Table 1:** Comparison of various modalities for decoding imagined speech.

**Method**	**Temporal resolution**	**Spatial resolution**	**Type**	**Portability**
EEG	0.06 ms^a^^,^^b^	25 mm^2^ (Yamazaki et al., 2013)	Non-invasive	Portable
MEG	0.1 ms^c^	1 mm (Singh, 2014)	Non-invasive	Non-portable
ECoG	0.02 ms^d^	4 mm (Muller et al., 2016)	Invasive	Portable
fMRI	500 ms (Yoo et al., 2018)	0.7 mm (Kashyap et al., 2018)	Non-invasive	Non-portable
fNIRS	100 ms (Metzger et al., 2017)	100 mm (Lu et al., 2010)	Non-invasive	Portable
ICE	3 ms (Ayodele et al., 2020)	0.05 mm (Ayodele et al., 2020)	Invasive	Portable

a *https://www.ant-neuro.com/products/eego_mylab/specs*.

b *The actual temporal and spatial resolution may be lower due to volume conduction effects (Burle et al., 2015)*.

c *https://www.compumedics.com.au/wp-content/uploads/2016/11/AH425-02-Orion-LifeSpan-MEG-brochure-JUNE-2019.pdf*.

d *https://www.gtec.at/product/gusbamp-research/*.

### 1.1. Inclusion/Exclusion Criteria

The primary source for the papers analyzed in this work was PubMed. Papers were selected for screening if their titles or abstracts included “imagined speech,” “covert speech,” “silent speech,” “speech imagery,” or “inner speech.” These keywords are wide enough to include all the works on imagined speech indexed in PubMed. This returned 504 results which were further screened for relevance. We discarded the papers that did not deal with decoding imagined speech, such as the papers on the manifestation of imagined speech in those suffering from various neurological disorders such as schizophrenia (for e.g., Livet and Salomé, 2020; Mitropoulos, 2020), global aphasia (GA) (for e.g., Sierpowska et al., 2020), and autism (for e.g., Mitsuhashi et al., 2018; Petrolini et al., 2020). It also included five review papers which are:

1. The review paper by Bocquelet et al. (2016) discusses the considerations in designing an imagined speech based BCI. Unlike our work, which focuses on EEG based speech BCI, the work by Bocquelet et al. is a review on the choice of brain region, decoding strategies in general, etc., with no particular reference to any data acquisition system such as fMRI, EEG, or ECoG.
2. The focused review article by Herff and Schultz (2016) compares the efficiency of different brain imaging techniques which can be used for decoding imagined speech from neural signals. This is significantly different from our paper, which reviews in-depth the methodological considerations in designing a system for decoding imagined speech from EEG.
3. The review articles by Martin et al. (2018), Rabbani et al. (2019), and Miller et al. (2020) deal exclusively with ECoG and no other modalities.

**Figure 1:**
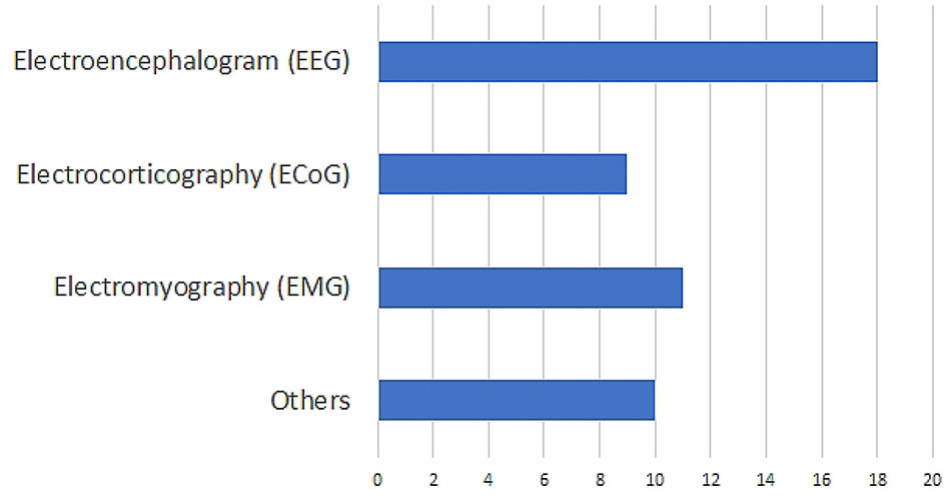
Distribution of the modalities used in the literature on decoding imagined speech. “Others” include functional magnetic resonance imaging (fMRI), functional near-infrared spectroscopy (fNIRS), intracortical electroencephalography (ICE) etc.

**Figure 2:**
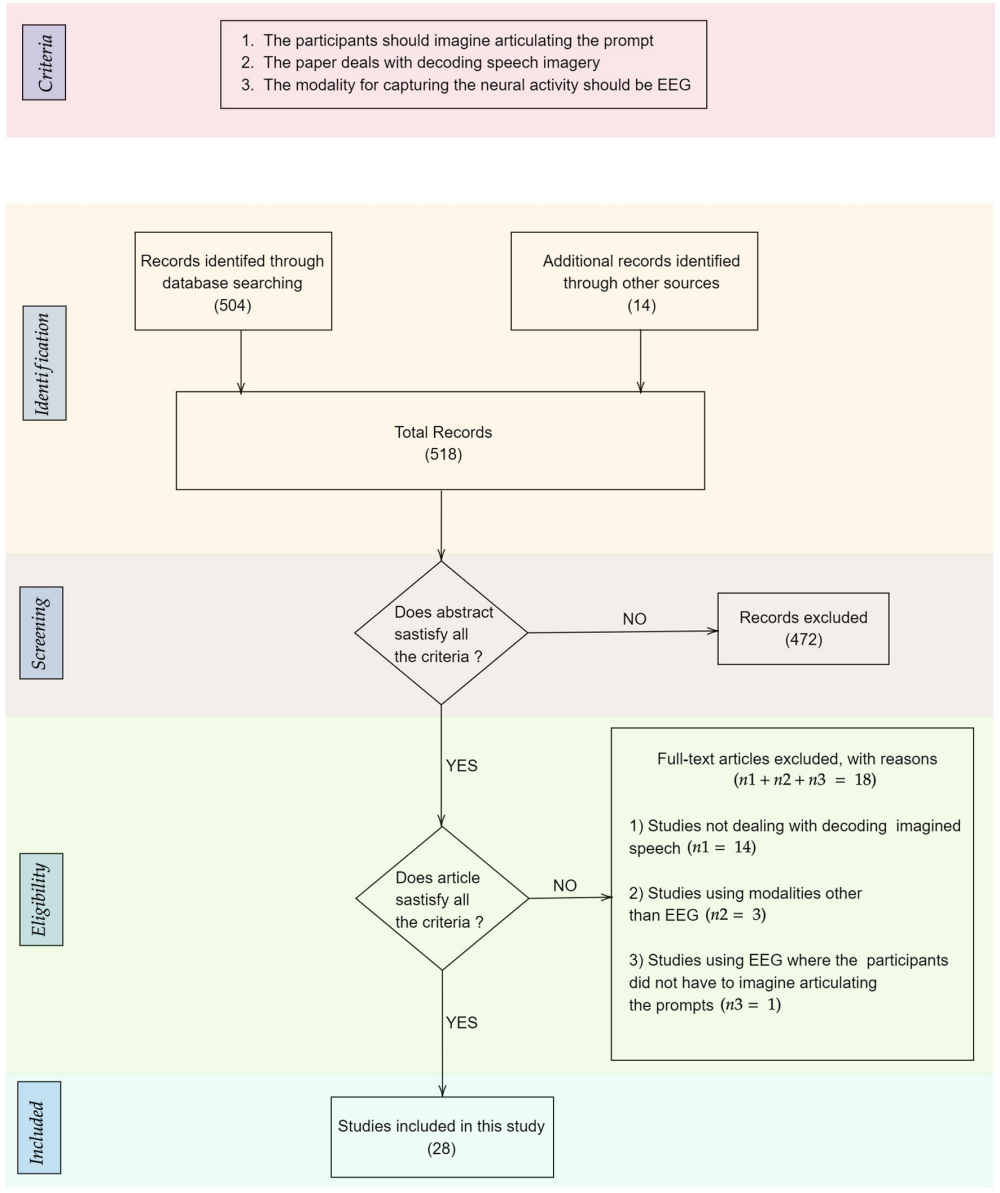
Flowchart detailing the database searches, the number of abstracts screened, the criteria applied for screening the papers, and the full texts retrieved. The number of records in each stage is given within parenthesis.

After this initial screening, we were left with 48 papers that deal with decoding imagined speech. The distribution of the modalities used for decoding imagined speech in these papers is given in Figure 1. These modalities include EEG, ECoG (Herff et al., 2015, 2016), fMRI (Yoo et al., 2004; Abe et al., 2011), fNIRS (Herff et al., 2012; Kamavuako et al., 2018; Sereshkeh et al., 2018), MEG (Destoky et al., 2019; Dash et al., 2020), ICE (Brumberg et al., 2011; Kennedy et al., 2017; Wilson et al., 2020) etc. Clearly, EEG is the most popular modality used for decoding imagined speech with 18 articles using it for capturing the neural changes during imagined speech. Among these 18 articles, the article by Imani et al. (2017) was not included since in the experimental protocol described in the article, the participants were not imagining articulating the prompts. In addition to the 17 papers indexed in PubMed, we selected 111 more relevant papers from other sources including IEEE Xplore and arXiv. A flowchart detailing the database searches, the number of abstracts screened and the full texts retrieved is shown in Figure 2. In addition to the 28 articles selected, several other articles were used as secondary sources for this paper. For instance, the section on the frequency band to be targeted for decoding imagined speech is based on articles on decoding imagined speech using ECoG.

**Figure 3:**
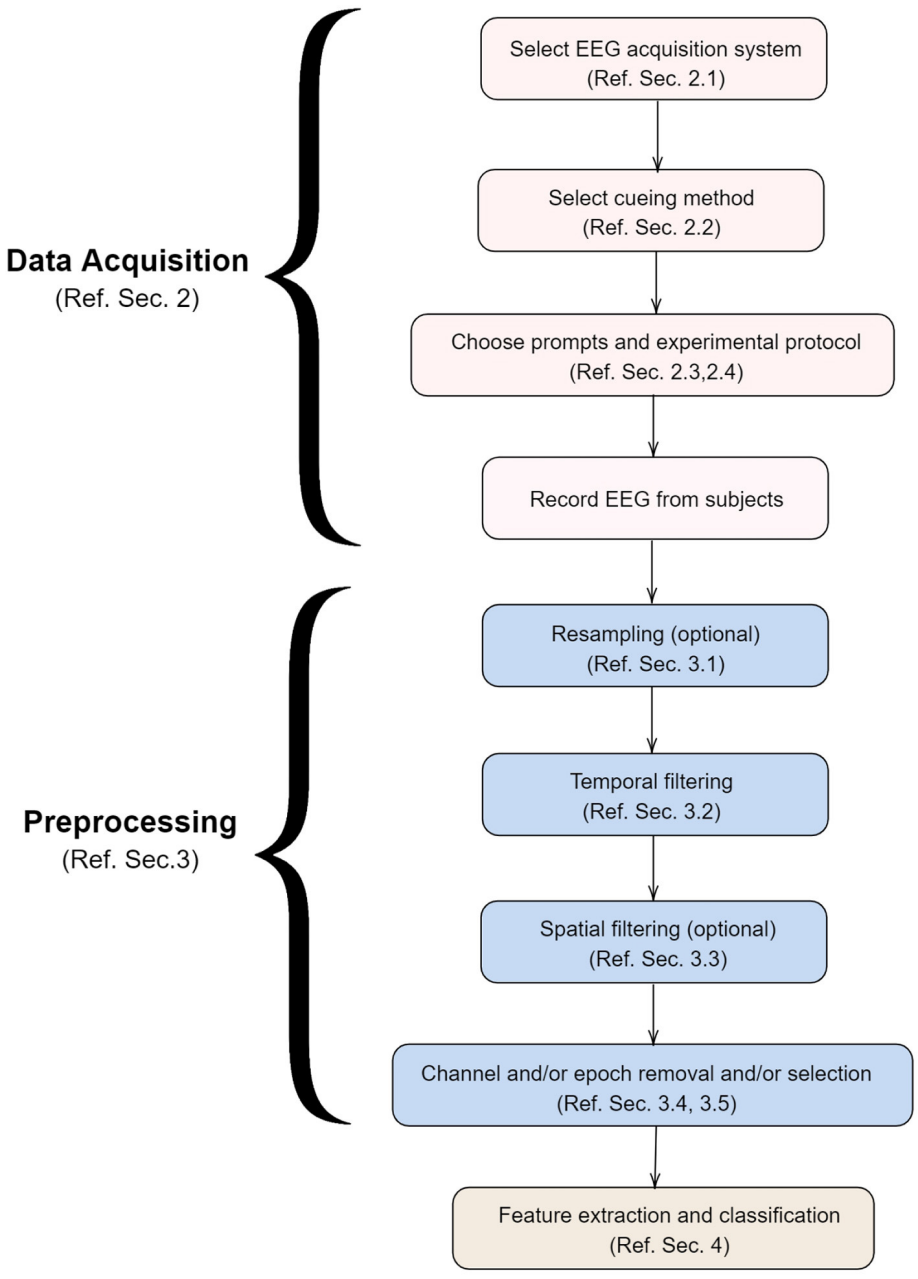
Various steps involved in the development of a system for decoding imagined speech from EEG. This paper is organized in the same order as above.

To the best of the knowledge of the authors, there is no review paper that focuses exclusively on EEG based systems for decoding imagined speech. The various factors involved in the development of such a system are shown in Figure 3 and discussed in detail in this paper in the same order. For the sake of completeness, we have also included a section on the neural correlates of imagined speech (section 1.2) and the types of BCI systems (section 1.3).

Specifically, the following are discussed in this paper:

- Neural correlates of imagined speech.
- Different categories of BCI systems.
- Methodological considerations that should be taken into account during data acquisition including the choice of prompts and stimulus delivery.
- Common preprocessing steps followed.
- Common feature extraction techniques and classification algorithms.
- Considerations in designing a speech imagery based online BCI system.
- Future directions in the field of BCI systems based on speech imagery neuro-paradigm.

### 1.2. Neural Correlates of Imagined Speech and Relationship with Articulated Speech

The prominent model for neural representation of articulated speech is the two-streams hypothesis (Hickok and Poeppel, 2007; Rauschecker and Scott, 2009). According to this, human beings have two distinct auditory pathways: ventral stream and the dorsal stream, both passing through the primary auditory cortex. In the ventral stream, phonemes are processed in the left superior temporal gyrus (STG) whereas words are processed in the left anterior STG (DeWitt and Rauschecker, 2012). Further, these region respond preferentially to speech than to semantically matched environmental sounds (Thierry et al., 2003). In the dorsal stream, auditory sensory representations are mapped onto articulatory motor representations. The information flows from primary auditory cortex into the pSTG and posterior superior temporal sulcus (STS). From there, it flows to left Sylvian parietal temporal (Spt). Further, the information moves to articulatory network 1 consisting of posterior inferior frontal gyrus (pIFG) and Brodmann area 44 (BA44) and articulatory network 2 consisting of primary motor cortex (M1) and ventral Brodmann area 6 (vBA6).

The relationship between the neural correlates of imagined speech and articulated speech is still a matter of debate. Two of the early hypotheses of neural correlates of imagined speech are due to Watson (1913), who argued that the neural correlates are similar and Vygotsky (1986), who argued that they are completely different. A large number of studies reported in the literature to verify these hypotheses are based on the speech production model proposed by Levelt (1993). The model splits articulated speech production into several phases such as (1) lemma retrieval and selection, (2) phonological code retrieval, (3) syllabification, (4) phonetic encoding and (5) articulation. The results of the studies based on Levelt's model are contradictory. Several studies (Bookheimer et al., 1995; Rosen et al., 2000; Palmer et al., 2001; Shuster and Lemieux, 2005) have shown that there is more activation in the motor and premotor areas (both lying in the frontal lobe) during articulated speech whereas some other studies (Huang et al., 2002; Basho et al., 2007) have shown that there is more activation in the frontal lobe during imagined speech. Thus, both Vygotsky's and Watson's hypotheses are not completely true.

Tracing a midline between Vygotsky's and Watson's hypotheses, Oppenheim and Dell (2008) proposed the surface-impoverished hypothesis. According to this hypothesis, imagined and articulated speech differ at the phonological level but have similar neural activation in the lexical level. This hypothesis is contradicted by several studies which show that the phonological and lexical features in both imagined and articulated speech are similar (Abramson and Goldinger, 1997; Brocklehurst and Corley, 2011; Corley et al., 2011). The current understanding is that Vygotsky hypothesis and the surface-impoverished hypothesis are partly true. A very recent study (Stephan et al., 2020) based on simultaneous application of both EEG and fNIRS has shown that imagined and articulated speech do differ at the phonological level (surface-impoverished hypothesis).

**Figure 4:**
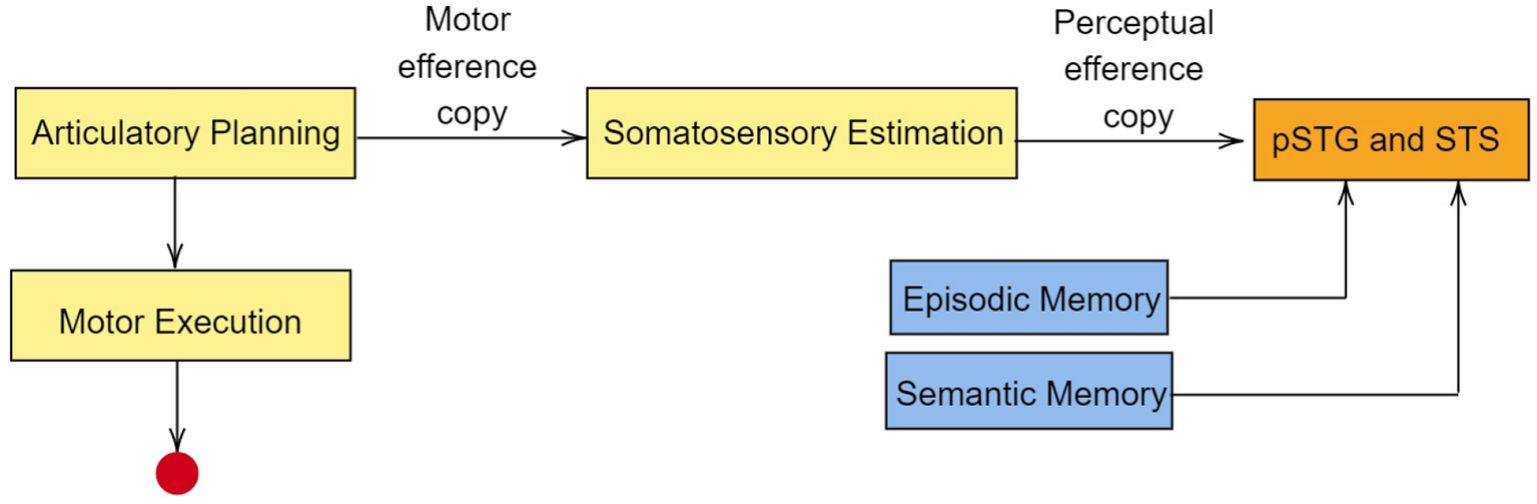
Simplified representation of dual stream prediction model (DSPM) for imagined speech. The dorsal stream is in yellow boxes, whereas the ventral stream is in blue boxes. The red circle represents the truncation of information at primary motor cortex in the case of speech imagery. pSTG, posterior superior temporal gyrus; STS, superior temporal sulcus. The primary auditory cortex lies in the superior temporal gyrus and extends into Heschl's gyri. Though Heschl's gyri is involved in speech perception, the region is not activated during speech imagery.

Based on MEG studies, Tian and Poeppel (2013) proposed a dual stream prediction model (DSPM) for imagined speech. This model is linked to the two-streams hypothesis. In DSPM too, two streams of information flow are present, the ventral stream and the dorsal stream. During speech imagery, articularotry planning occurs in premotor cortex. Since motor movements are not intended during speech imagery, the information flow is terminated at M1 (Tian and Poeppel, 2012). Nevertheless, a motor efference copy is sent to inferior parietal cortex for somatosensory estimation (Whitford et al., 2017). The perceptual efference copy generated at the inferior parietal cortex is sent to pSTG (posterior superior temporal gyrus) and STS (superior temporal sulcus). The idea of efference copy in speech imagery was proposed as a result of magnetoencephalography studies by Tian and Poeppel (2010). In the MEG recordings, an activation in the auditory cortex was observed immediately after speech imagery. Since there is no overt auditory feedback during speech imagery, the observed activation in the auditory cortex was explained using the possible existence of an internal forwarding model deploying efferent copies in the auditory cortex. According to Tian and Poeppel, the neural signal generated during articulation preparation is used to predict the anticipated auditory signal in speech imagery, via a time-locked auditory efferent copy, which causes the observed activity in the auditory cortex. In the ventral stream, auditory representation is sent to pSTG and STS. Along with this auditory representation, the ventral stream also retrives episodic memory and semantic from middle temporal lobe (MTL) and posterior middle temporal gyrus (pMTG) respectively. A pictorial representation of this model is given in Figure 4. The primary auditory cortex contains regions such as pSTG and Heschl's gyri (transverse temporal gyri). Lu et al. (2021) have shown that although Heschl's gyri is involved in speech perception, the region is not activated during speech imagery.

Results of many neuroimaging, behavioral and electrophysiological studies such as Tian et al. (2016, 2018), Whitford et al. (2017), Lu et al. (2021) also support the presence of efference copies in imagined speech. Functional MRI studies by Tian et al. (2016) revealed greater activation in the frontal-parietal sensorimotor regions, including sensorimotor cortex, subcentral (BA 43), middle frontal cortex (BA 46) and parietal operculum (PO) during speech imagery. This observed activation is similar to the activation pattern corresponding to articulation preparation (Brendel et al., 2010; Price, 2012). Thus, the brain activity pattern corresponding to speech imagery is due to articulation preparation including motor planning and the activation of the auditory cortex due to efference copies.

### 1.3. Types of BCI Systems

#### 1.3.1. Online vs. Offline BCI Systems

In offline BCI systems, such as the systems described in Park et al. (2012), Edelman et al. (2015), Khan and Hong (2017), and Tayeb et al. (2019) the EEG data acquired from the participant is not processed in real-time; rather it is processed at a later stage. This approach is useful only in a research environment but gives the researchers the freedom to use computationally expensive algorithms for processing the EEG data. On the other hand, in an online BCI system, such as the systems described in Lal et al. (2005), Bin et al. (2009), Hazrati and Erfanian (2010), Gui et al. (2015), Mondini et al. (2016), Wu (2016), and Khan and Hong (2017), the EEG data is processed in real-time giving real-time BCI outputs. This places an upper limit on the computational complexity of the algorithms used but has significant practical application; rather, a BCI system is practically useful only if it can be translated to an online system. Most of the works on decoding imagined speech employ offline strategies except for the work by Sereshkeh et al. (2017b) in which EEG is used and the others which make use of functional near-infrared spectroscopy (fNIRS) (Gallegos-Ayala et al., 2014; Naseer et al., 2014; Sereshkeh et al., 2018). The systems described in Gallegos-Ayala et al. (2014), Naseer et al. (2014), Sereshkeh et al. (2017b) have two degrees of freedom, whereas the system described in Sereshkeh et al. (2018) has three degrees of freedom.

#### 1.3.2. Exogenous vs. Endogenous BCI Systems

In an exogenous (*exo:* outside or external, *genous*: producing) BCI system, external stimulus is used for generating distinct neural activation such event-related potentials (ERP) such as P300 and evoked potentials such as steady state visually evoked potentials (SSVEP). On the other hand, in an endogenous (*endo:* inside or internal, *genous*: producing) BCI system, the neural activation is not because of any external stimuli. In an endogenous BCI, motor imagery, speech imagery etc. can be used for eliciting the required neural activation. Graz BCI (Müller-Putz et al., 2016) is an endogenous BCI system whereas Unicorn speller (Al-Nuaimi et al., 2020) is an exogenous BCI system.

#### 1.3.3. Synchronous vs. Asynchronous BCI Systems

In a synchronous BCI, the EEG capture for analysis is synchronized with a cue. That is, in case of speech imagery based BCI system, the time window for imagination is predefined and any EEG captured outside this window is discarded. In an asynchronous BCI, the capture of neural activity is not linked to any cues. Though asynchronous BCI is a more natural mode of interaction, the BCI system will be more complex since it has to decide whether the ellictted neural activity is because of an intentional mental activity from the subject or because of an unintentional mental activity.

## 2. Data Acquisition

### 2.1. Type of EEG Acquisition System

Most of the researchers, including Zhao and Rudzicz (2015), Min et al. (2016), Nguyen et al. (2017), Koizumi et al. (2018), and Sereshkeh et al. (2017a) have used a 64-electrode EEG system with a sampling rate of 1 KHz for acquiring the EEG data corresponding to imagined speech. In the case of the work reported by Deng et al. (2010) and Brigham and Kumar (2010), 128-electrode EEG data has been recorded at a sampling rate of 1 KHz. Wang et al. (2013) and García et al. (2012) have used lesser number of EEG channels. Wang et al. have used two different electrode configurations: a 30-electrode system covering the entire head and a 15-electrode system covering only the Broca's and Wernicke's areas. The signal sampling rate is 250 Hz in both the cases. Jahangiri et al. have used a 20-electrode EEG system with a sampling rate of 500 Hz in Jahangiri et al. (2018) and a 64-electrode EEG system with a sampling rate of 2048 Hz in Jahangiri and Sepulveda (2019), Jahangiri et al. (2019). Watanabe et al. (2020) have used a 32-electrode EEG system with a sampling rate of 1 KHz. A 64-electrode EEG system has been used in Zhang et al. (2020) with a sampling rate of 500 Hz.

Though most of the researchers have made use of high-density EEG systems, the approach of Wang et al. in using only the channels covering the Broca's and Wernicke's areas has the following advantages:

1. Studies based on common spatial patterns (CSP) and event-related spectral perturbation (ERSP), reported in Wang et al. (2014), Nguyen et al. (2017), and Zhao and Rudzicz (2015), have shown that the most significant EEG channels for classifying speech imagery are the ones covering the Broca's and Wernicke's areas.
2. When a brain-computer interface (BCI) system is deployed for practical applications, it is better to have as minimum a number of EEG channels as possible. This is because EEG systems with less number of channels are cheaper and can be more easily setup and maintained than high-density systems.

However, the extent of involvement of Broca's and Wernicke's areas in language processing is still a point of contention (Binder, 2015; Tremblay and Dick, 2016). Modern neuroimaging studies have shown that in addition to Broca's and Wernicke's areas, other areas in the temporal lobe are also involved in language processing (Poeppel et al., 2008; Newman et al., 2010). Hence, though using only the EEG channels covering the Broca's and Wernicke's areas has certain practical advantages, there is a trade-off in terms of the information captured (Srinivasan et al., 1998). Also, when independent component analysis (see section 3.4) is used, higher the number of channels, better is the decomposition, although there is a ceiling in the quality of decomposition when the number of channels reaches 64 (Klug and Gramann, 2020).

With respect to commercial grade and research grade EEG acquisition devices, more than 20% of the studies reviewed in this article make use of commercial grade devices, characterized by low EEG density and/or low sampling rate. Though there can be a detrimental effect in the quality of the EEG signal acquired, commercial grade systems are closer to a practical BCI system in terms of cost of the device. Additionally, devices such as ENOBIO (Ruffini et al., 2007) and Emotiv (Duvinage et al., 2012) used by Jahangiri et al. (2018) and García et al. (2012) respectively offer a setup time of less than 5 min.

**Figure 5:**
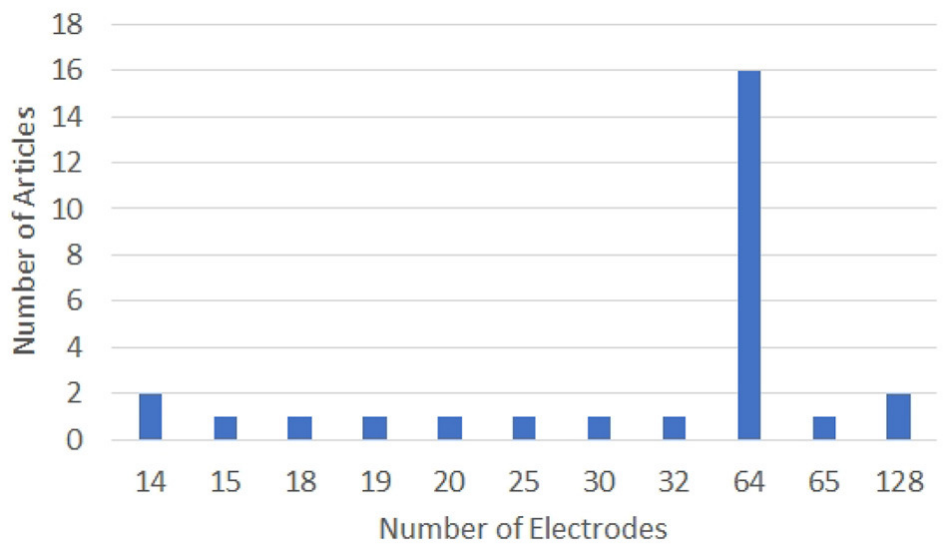
Graph showing the number of electrodes used for data acquisition in various works on decoding imagined speech from EEG. X and Y-axes represent the number of electrodes and articles, respectively.

**Figure 6:**
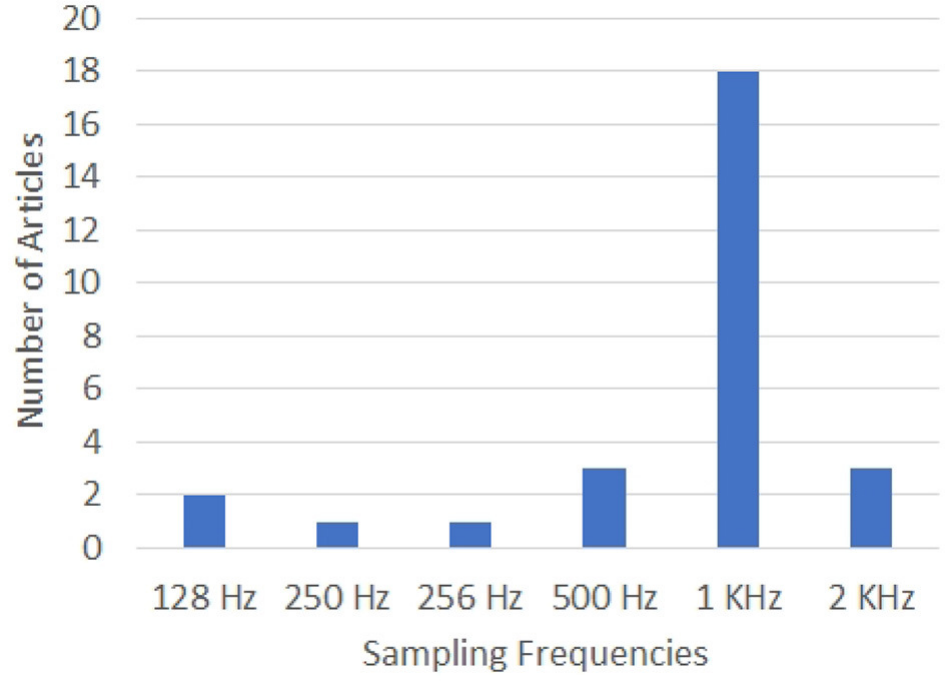
Graph showing the sampling rates used for data acquisition by the various works in the literature on decoding imagined speech from EEG. X-axis gives the sampling rates and Y-axis gives the number of articles using each specific sampling frequency.

The configurations of the EEG systems used in the articles analyzed in this work are given in Figures 5, 6. Clearly, 64-electrode EEG system with the sampling rate of 1 KHz is the most popular configuration of the EEG systems used for data acquisition.

A comparison of the types of EEG systems, sampling rate, decoding strategy and the maximum number of degrees of freedom of various studies reviews in this work is given in Table 2.

**Table 2:** Comparison of the types of EEG systems, sampling rate, decoding strategy and maximum number of degrees of freedom of various studies reviewed in this work.

**Sl. No**.		**Type of EEG system**	**Sampling rate**	**Resampled frequency**	**Decoding strategy**	**Maximum number of degrees of freedom reported**
1	Jahangiri et al. (2019)	Research	2 KHz	256 Hz	Offline	4
2	Wang et al. (2013)	Research	250 Hz	N/A	Offline	2
3	Jahangiri et al. (2018)	Commercial	500 Hz	256 Hz	Offline	4
4	Tøttrup et al. (2019)	Commercial	500 Hz	N/A	Offline	6 (including two motor imagery)
5	Saha et al. (2019b)	Research	1 KHz	N/A	Offline	2
6	Koizumi et al. (2018)	Research	1 KHz	N/A	Offline	12 (including six visual imagery)
7	Sereshkeh et al. (2017a)	Research	1 KHz	N/A	Offline	2
8	Deng et al. (2010)	Research	1 KHz	N/A	Offline	6
9	Zhang et al. (2020)	Research	500 Hz	N/A	Offline	4
10	Cooney et al. (2020)	Commercial	1 KHz	N/A	Offline	6
11	Chengaiyan et al. (2020)	Commercial	256 Hz	N/A	Offline	5
12	Brigham and Kumar (2010)	Research	1 KHz	N/A	Offline	2
13	Cooney et al. (2018)	Research	1 KHz	N/A	Offline	11
14	Pawar and Dhage (2020)	Research	1 KHz	N/A	Offline	4
15	Nguyen et al. (2017)	Research	1 KHz	256 Hz	Offline	3
16	Sereshkeh et al. (2017b)	Research	1 KHz	N/A	Online	2
17	Watanabe et al. (2020)	Research	1 KHz	N/A	Offline	3
18	Jahangiri and Sepulveda (2017)	Research	2 KHz	256 Hz	Offline	4
19	Jahangiri and Sepulveda (2019)	Research	2 KHz	256 Hz	Offline	4
20	García et al. (2012)	Commercial	128 Hz	N/A	Offline	5
21	Min et al. (2016)	Research	1 KHz	250 Hz	Offline	2
22	Saha and Fels (2019)	Research	1 KHz	256 Hz	Offline	3
23	Saha et al. (2019a)	Research	1 KHz	N/A	Offline	2
24	Panachakel et al. (2020a)	Research	1 KHz	256 Hz	Offline	2
25	Panachakel et al. (2019)	Research	1 KHz	N/A	Offline	11
26	García-Salinas et al. (2019)	Commercial	128 Hz	N/A	Offline	5
27	Cooney et al. (2019)	Commercial	1 KHz	128 Hz	Offline	5
28	Balaji et al. (2017)	Research	250 Hz	N/A	Offline	4

### 2.2. Mode of Stimulus Delivery

Three methods have been primarily followed by researchers to cue the participant as to what the prompt is and when to start imagining speaking the prompt. These are (1) auditory (Brigham and Kumar, 2010; Deng et al., 2010; Min et al., 2016; Koizumi et al., 2018); (2) visual (Wang et al., 2014; Sereshkeh et al., 2017a; Jahangiri et al., 2018; Koizumi et al., 2018); and (3) a combination of auditory and visual cues (Zhao and Rudzicz, 2015; Coretto et al., 2017; Nguyen et al., 2017; Watanabe et al., 2020). Although somatosensory cues have been used for motor imagery (Panachakel et al., 2020b), no such work has been reported for speech imagery.

Since both Broca's and Wernicke's areas are involved in imagined speech (Hesslow, 2002), it is difficult to remove the signature of the auditory cue from the EEG signal recorded during speech imagery. It has been shown that visual cues elicit responses in the occipital lobe (Nguyen et al., 2017). Since the occipital lobe is involved neither in production nor comprehension of speech, discarding the EEG channels over the occipital lobe eliminates the interference of the visual cue on the EEG recorded during imagined speech. Hence, the use of visual cues obviates the preprocessing steps for removing auditory cues. Although studies (Ikeda et al., 2012) have shown that the excitation of the primary motor cortex is higher when auditory and visual cues are used, the practical benefit of such a system, especially in the field of rehabilitation is limited. This is also true for the use of somatosensory stimuli in motor imagery as in Panachakel et al. (2020b).

### 2.3. Repeated Imagination During a Trial

In most of the works, the participant is supposed to imagine speaking the prompt only once. However, in a few works such as Brigham and Kumar (2010), Deng et al. (2010), Nguyen et al. (2017), Koizumi et al. (2018), the participants are asked to imagine speaking the prompt multiple times in the same trial. In all these works, auditory clicks are provided during each trial to make the participants have a sense of rhythm at which the prompt should be imagined. Nguyen et al. have used this periodicity in imagination to identify the channels that have the most information corresponding to the cortical activity of speech imagery. They have computed the autocorrelation functions of all the EEG channels and applied discrete Fourier transform (DFT) on the computed autocorrelation functions. The channels were graded based on the proximity of the highest peak of the frequency spectrum to the frequency at which the auditory cues were provided. It was observed that the channels covering Broca's area, Wernicke's area and motor cortex had the highest peaks in the frequency spectrum closer to the frequency of the auditory cues. Hence, multiple imagination can be used to check the quality of the acquired data, as carried out by Nguyen et al.

Unlike the approach by Nguyen et al. and Brigham et al., Deng et al.'s approach required the participants to imagine the prompts in three different rhythms. They have shown that in addition to the imagined prompt, the rhythm at which the prompt is imagined can also be decoded from the recorded EEG signal.

In our own experiments reported in Panachakel et al. (2020b), we have observed that the EEG signatures become more prominent across multiple imaginations in the same trial but deteriorate across multiple trials in the same recording session.

**Figure 7:**
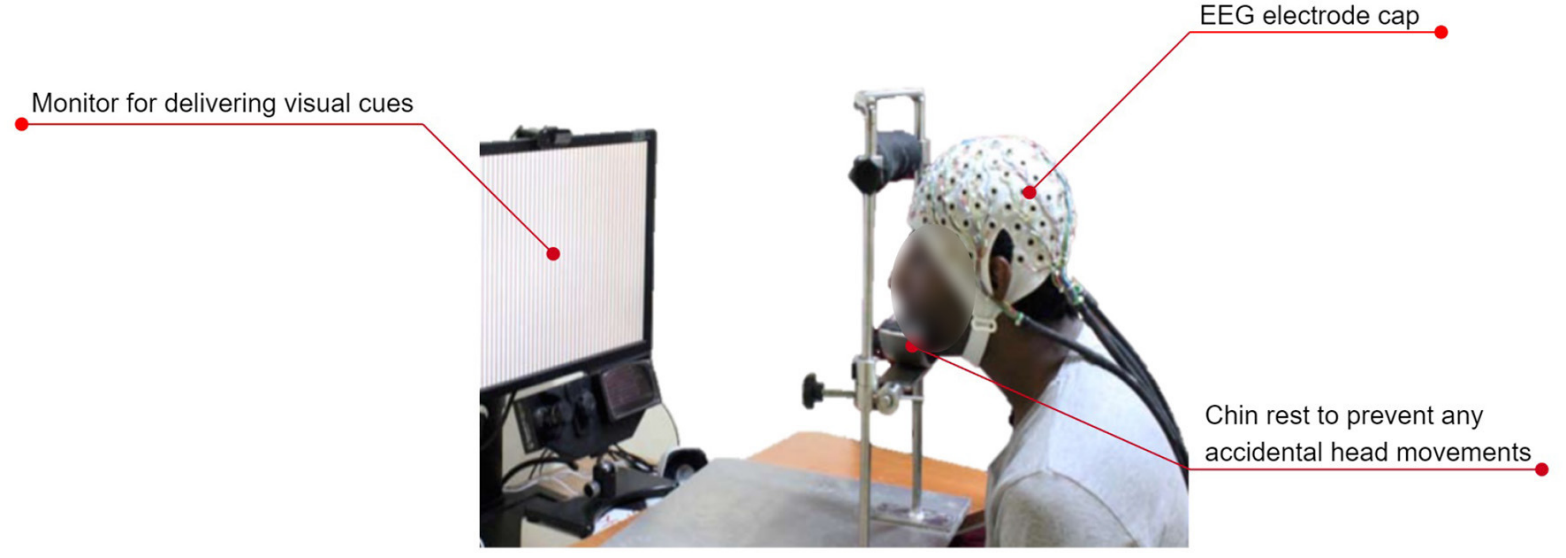
A typical experimental setup used for recording EEG during speech imagery. The subject wears an EEG electrode cap. A monitor cues the subject on the prompt that must be imagined speaking. An optional chin rest prevents artifacts due to unintentional head movements. Figure adapted with permission from Prof. Supratim Ray, Centre for Neuroscience, Indian Institute of Science, Bangalore.

Figure 7 shows the typical experimental setup followed by most of the researchers.

### 2.4. Choice of Prompts

#### 2.4.1. Syllables Only

Min et al. (2016) have used the vowel sounds /a/, /e/, /i/, /o/, and /u/ as the prompts. These sounds are acoustically stationary, emotionally neutral and easy to imagine uttering. Nevertheless, it is shown in Nguyen et al. (2017) that prompts with higher complexity (more number of syllables) yield higher classification results in decoding imagined speech [more details about Nguyen et al. (2017) are given in the following sections]. They have also shown that distinct prompts with different levels of complexity (such as one monosyllabic word and one quadrisyllabic word) yield further improvement in the accuracy. The dataset developed by Brigham and Kumar (2010) has only two prompts /ba/ and /ku/. The reason for the choice of these prompts is the difference in their phonological categories:

1. /ku/ has a back vowel, whereas /ba/ has a front vowel
2. /ba/ has a bilabial stop, whereas /ku/ has a gutteral stop.

Deng et al. (2010) also used the syllabic prompts /ba/ and /ku/. Contrary to the approach by Brigham et al., the participants in Deng et al.'s work were instructed to imagine the prompts in three different rhythms in different trials. The cue for rhythm was given using auditory clicks. They have shown that it is possible to decode the rhythm from the imagined EEG, even when the algorithm failed to decode the imagined syllable.

In the works by Jahangiri et al. (2018, 2019) and Jahangiri and Sepulveda (2019), four syllables, namely /ba/, /fo/, /le/, and /ry/ were chosen as the prompts. These syllables were chosen since they were phonetically dissimilar. It is shown by Cummings et al. (2016) that phonetically dissimilar prompts create distinct neural activities. In Jahangiri and Sepulveda (2019), the prompt to be imagined is cued using auditory cues whereas in Jahangiri et al. (2019) and Jahangiri et al. (2018), visual cues are used. In Jahangiri et al. (2018) the participants are cued by showing arrows in four different directions, where each direction corresponds to a specific phonemic structure the subject needs to imagine. For example, left arrow corresponds to the prompt /le/ whereas right arrow corresponds to the prompt /ry/. In Jahangiri et al. (2019), the prompt to be imagined is cued using the game “whack-a-mole.” The subject needs to imagine the location of the hole from where the mole appeared in the game and the recorded EEG is used for decoding the imagined word.

In Watanabe et al. (2020), three prompts are used, all formed using the syllable /ba/. Each prompt consisted of three /ba/ and two /ba:/, uniform duration of 1800 ms and uniform pitch height of 200 Hz.

#### 2.4.2. Words Only

In the protocol followed by Sereshkeh et al. (2017a), the participants were to imagine the response (yes/no) to several perceptual, non-emotive binary questions like “Is the word in uppercase letters? WORD.” These two English words were chosen due to the following reasons:

1. Their relevance in BCI applications for patients who cannot communicate in any other way.
2. /y/ and /n/ differ in the place and manner of articulation. Zhao and Rudzicz (2015) have shown that these differences in the place and manner of articulation are captured by the EEG signals.
3. The vowels /e/ and /o/ originate in different areas of the tongue and hence might have differentiable EEG signatures (Mugler et al., 2014).

In the work by Balaji et al. (2017), bilingual prompts were used. Specifically, “yes” and “no” in English and “Haan” and “Na” in Hindi (meaning “yes” and “no” respectively) were used. Similar to Sereshkeh et al. (2017a), the experimental protocol required the participants to imagine the response to several binary questions, either in English or Hindi. They have reported an accuracy of 85.2% when decision was decoded from the recorded EEG and an accuracy of 92.18% when the language was decoded, clearly indicating that bilingual prompts have higher potential for being suitable prompts for imagined speech.

In the work by García et al. (2012), five Spanish words were used as the prompts. The words were “arriba,” “abajo,” “izquierda,” “derecha,” and “seleccionar.” The equivalent English words are “up,” “down,” “left,” “right,” and “select,” respectively. In the work by Koizumi et al. (2018) six Japanese words (“ue,” “shita,” “hidari,” “migi,” “mae,” and “ushiro”) were used as the prompts, meaning “up,” “down,” “left,” “right,” “forward,” and “backward,” in English. These words were chosen because the words correspond to instructions a user might use for controlling a computer cursor or a wheelchair. In a very recent work by Pawar and Dhage (2020), a similar set of prompts was used. Pawar and Dhage used the prompts “left,” “right,” “up,” and “down.” This choice of prompts is not only motivated by the usefulness of these prompts in practical applications but also because of their diverse manner and places of articulation.

In Chengaiyan et al. (2020), 50 consonant-vowel-consonant words were used as the prompts. All the five vowels were considered and for each vowel, 10 words were used. One of the aims of the study was to classify vowels and these words were chosen since each word has only one vowel. This choice of prompts extends the study by several other authors in classifying vowels using imagined speech EEG.

#### 2.4.3. Both Words and Syllables

The two prominent datasets having both syllable and word prompts are the datasets developed by Zhao and Rudzicz (2015) and Coretto et al. (2017). The dataset by Zhao et al. consists of seven monosyllabic propmts, namely /iy/, /uw/, /piy/, /tiy/, /diy/, /m/, /n/, and four words “pat,” “pot,” “knew,” and “gnaw.” Here, “pat” & “pot” and “knew” & “gnaw” are phonetically-similar pairs. These prompts were chosen to have the same number of nasals, plosives, and vowels, as well as voiced and unvoiced phonemes.

Similar to the dataset by García et al. (2012), the dataset by Coretto et al. also consisted of six Spanish words which are “arriba,” “abajo,” “derecha,” “izquierda,” “adelante,” and “atr‘as.” The equivalent English words are “up,” “down,” “right,” “left,” “forward,” and “backward,” respectively. In addition to these six prompts, the vowels /a/, /e/, /i/, /o/, and /u/ were also used as prompts.

Nguyen et al. (2017) collected imagined speech data using four different types of prompts, namely short words, long words, short-long words, and vowels. The three vowels used as prompts were /a/, /i/, and /u/. The shorts words used were “in,” “up,” and “out,” all of which are monosyllabic. The long words used are “independent” and “cooperate”, both having four syllables with none of the four syllables common between them. Nguyen et al. performed one more experiment in which the prompts were “in” (monosyllabic) and “cooperate” (quadrisyllabic). The aim of the experiment was to find out whether the difference in the length of the prompt had any effect on the decoding of imagined speech. As mentioned in section 2.4.1, the authors have reported an improvement in accuracy when prompts of different lengths are used.

#### 2.4.4. Lexical Tones

In some languages (known as tonal languages), pitch is used to differentiate lexical or grammatical meaning (Myers, 2004). One such tonal language is Mandarin where the minimal tone set consists of five tones. Out of these five lexical tones, four tones (flat, rising, falling-rising, and falling) are used with the syllable /ba/ in Zhang et al. (2020). This is the only work in decoding imagined speech where lexial tones are used as prompts.

Five commonly used prompts and their significance are given in Table 3. We have only listed the common prompts used in multiple articles. Prompts which are not used in multiple articles are not listed.

**Table 3:** Five common prompts used in decoding imagined speech and their significance.

**Sl. No**.	**Prompt**	**Significance**
1	/ba/, /fo/, /le/ and /ry/	Differences in place and manner of articulation.
2	“up”, “down”, “left” and “right”	Useful in controlling a computer mouse.
3	“yes” and “no”	Differences in place and manner of articulation, useful in responding to binary questions.
4	/a/, /e/, /i/, /o/ and /u/	Acoustic stationarity, differences in place and manner of articulation.
5	“in” and “cooperate”	Difference in complexity.

*Prompts which are not common in the literature are not tabulated here*.

## 3. Preprocessing

### 3.1. Resampling

Prior to preprocessing, some researchers employ a downsampler to resample the EEG data to a lower sampling rate. This is carried out in order to reduce the computational complexity involved in processing the data. Depending on how the features are extracted, this can also help ameliorate the problems associated with high dimensional feature vectors commonly referred to as the “curse of dimentionality.” Brigham and Kumar (2010), Nguyen et al. (2017), and Min et al. (2016) resampled the data from 1 KHz to 256 Hz during preprocessing making the data more manageable.

### 3.2. Temporal Filtering

In the task of classification of motor imagery, researchers mostly agree on the frequency band to be targeted for the best performance but in the case of imagined speech, this consensus is absent. Quite often, the frequency band is decided based on the type of the artifacts present in the recorded signal and how they are removed. Most of the works consider the frequency band from 8 to 20 Hz. In addition to this band, frequency band from 2 to 50 Hz is also used in several works. In all the articles reviewed in this work, only seven works use the frequency band above 80 Hz and out of these seven, only five works (Jahangiri et al., 2018, 2019; Koizumi et al., 2018; Jahangiri and Sepulveda, 2019; Pawar and Dhage, 2020) use frequency band from 80 to 100 Hz.

Jahangiri et al. have used the entire frequency range up to 128 Hz except for the narrow band from 49.2 to 50.8 Hz to remove the line noise whereas Koizumi et al. (2018) have used the frequency range from 1 to 120 Hz and have reported a higher classification accuracy when features extracted from the high gamma band (60–120 Hz) are used. Pawar and Dhage (2020) have used the frequency range from 0.5 to 128 Hz. Jahangiri et al. have supported the use of this band based on the high gamma activity observed in electrocorticography (ECoG) data recorded during imagined speech tasks (Greenlee et al., 2011; Llorens et al., 2011) and have reported higher activity in the band 70 to 128 Hz during imagination of the prompts.

**Figure 8:**
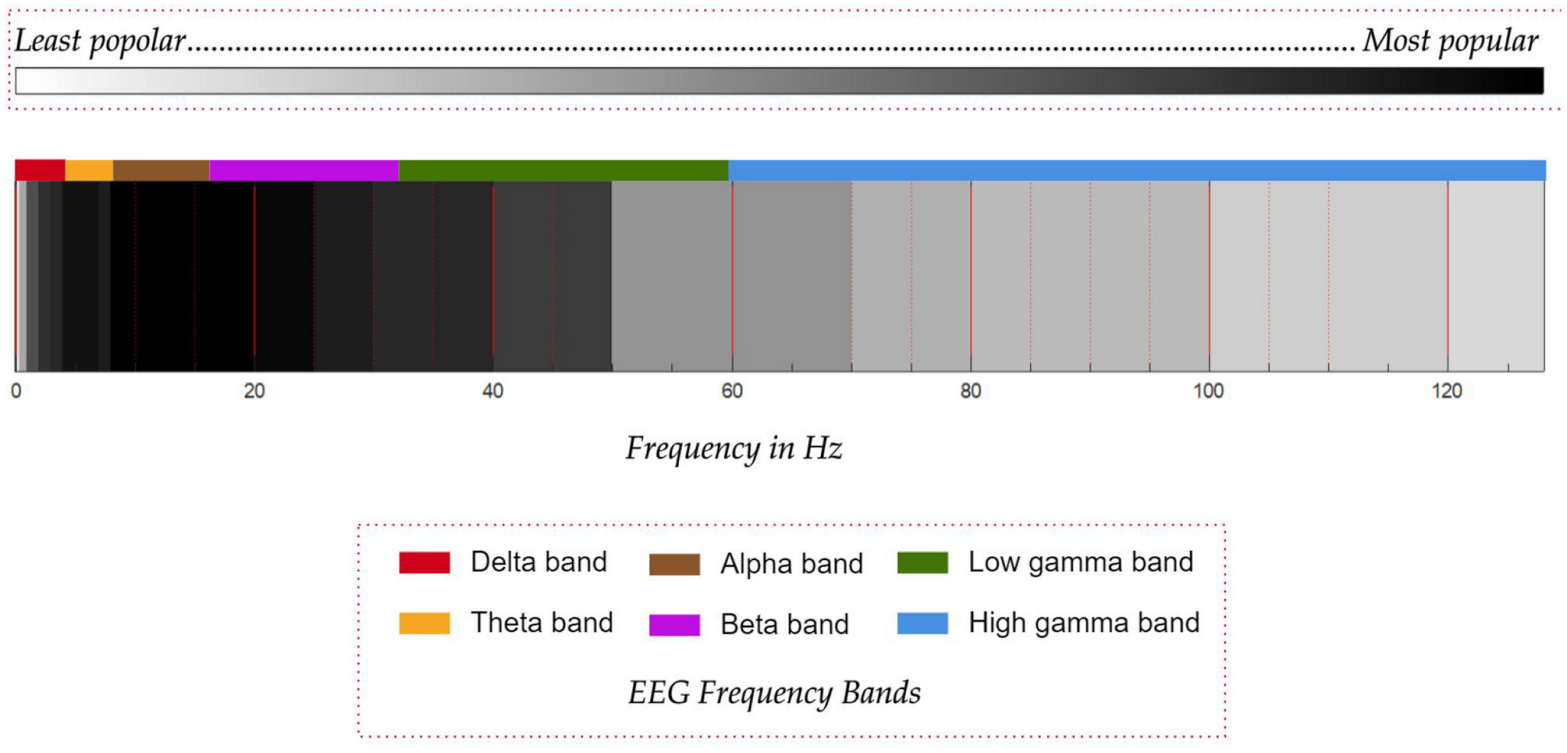
Comparison of the popularity of frequency bands used in works on decoding imagined speech from EEG. Darker shades of black represent more popular frequency bands. Common EEG frequency bands are given in different colors.

However, there are also studies in the literature (Whitham et al., 2007; Muthukumaraswamy, 2013) which have shown that the high gamma activity observed in EEG signals may be due to muscular artifacts. Moreover, it has been shown by Whitham et al. (2008) that imagination induces muscular artifacts in the EEG recorded from normal subjects. Thus, more focused studies are required as shown by Boytsova et al. (2016) to understand the reliability of high-gamma band activity observed in EEG, where muscular activities are suppressed using muscle relaxants. In fact, Koizumi et al. (2018) themselves have speculated in their work that the higher accuracy with the use of high gamma band might be due to EMG artifacts. It may be noted that the contention is only on the high-gamma activity observed in EEG and not in ECoG. A graphical comparison of the frequency bands used in the various works in the literature is given in Figure 8. The reduced use of gamma band compared to the lower frequency bands is probably on account of the uncertainty of the gamma band in EEG. The other important factor is that the EEG power spectrum follows a 1/*f* power law, which means that the power in the gamma band reduces with increasing frequency, thus decreasing the signal-to-noise ratio. From the work by Synigal et al. (2020), it is clear that it is the envelope of the EEG gamma power, and not the EEG itself that is well-correlated with the speech signal. Thus, this indicates that the gamma band may have issues of low signal-to-noise ratio.

Section 4.4 compares the performance of the systems proposed by Koizumi et al. (2018) and Pawar and Dhage (2020) based on Cohen's kappa value.

### 3.3. Spatial Filtering

Most works do not employ any spatial filtering in the preprocessing. The only exceptions are the works by Zhao and Rudzicz (2015) and Cooney et al. (2018), who used a narrow Laplacian filter. A Laplacian filter uses finite difference to approximate the second derivative. In the case of a highly localized Laplacian filter, the mean of the activities of the four nearest channels is subtracted from the central channel [refer McFarland et al. (1997) for more details on Laplacian filters used in EEG processing]. Spatial filtering is generally avoided in the preprocessing since Laplacian filter is a high pass filter, which may reduce the amount of useful information in the signal (Saha et al., 2019b).

### 3.4. Channel/Epoch Rejection

EEG signals are almost always corrupted by electrical potentials generated by ocular and facial muscles. Since the amplitude of EEG is very small (in the order of μ*V*) compared to the EMG generated by the muscles (in the order of *mV*), it is important to remove these artifacts from the EEG signal. It is difficult to remove these artifacts and methods based on heuristics are often combined with signal processing algorithms such as BSS (blind source separation) and employed for this purpose. ICA (independent component analysis) is the most common BSS algorithm used for preprocessing EEG and hence it is discussed in some detail in this section.

Let *X* be a matrix containing the set of *M* samples each of *N* observed signals (individual EEG channels in our case). In other words each of the *N* signals {*x*_1_(*t*), *x*_2_(*t*), …, *x*_*N*_(*t*)} is arranged as one of the columns of *X* and each column has *M* samples of the corresponding channel. Thus, the dimension of *X* is *M* × *N*. To put into the perspective of EEG signal processing, suppose EEG signal is acquired using a 64-channel EEG system with common average referencing at the sampling rate of 1,024 Hz for a duration of 10*s*, then the dimension of *X* used for storing this EEG will be 10240 × 64. These *N* observed signals are generated from a set of *K* source vectors (where *K* ≤ *N*) as given below:

(1) $$\begin{array}{l} X=SA \end{array}$$

where *S* is a *M* × *K* matrix containing the source signals that generated the observed signals in *X* and *A* is called the mixing matrix of dimension *K* × *N*. This linear model is consistent with the physics of EEG (Parra et al., 2005). Specifically, the i-th column of *X* is obtained as

(2) $$\begin{array}{l} X_{i}=Sa_{i} \end{array}$$

where *a*_*i*_ denotes the *i*th column of *A*. Our goal is to find the unmixing matrix, *W* = *A*^−1^ so that we can obtain the sources which generated the observed signals. One motivation for finding the sources is for denoising or removing noise from the observed signal. Noise is a relative term used to refer to any signal that is undesirable in the given context. For instance, if we are trying to decode imagined speech from EEG, information about eye blinks is not useful and electrical activity generated by the extraocular muscles is considered as a noise signal although in the context of a BCI system that relies on eye blinks, this signal carries information. ICA is the most commonly used method for removing these artifacts (Jiang et al., 2019). ICA essentially tries to identify the source of the eye blink and this source is suppressed to remove eye blink artifacts from the recorded EEG signal. Once we find out *W*, the unmixing or demixing matrix, the sources can be obtained from the observed signals by using the following relation:

(3) $$\begin{array}{l} S=XW \end{array}$$

Clearly, it is impossible to find a unique *W* using only *X* and hence we employ some measures that the sources should satisfy. The measure or cost is selected in such a way that the sources are statistically independent of each other. This intuitively makes sense, since the sources responsible for generating the EEG signals corresponding to imagined speech are independent of the extraocular muscles that generate the electrical activity corresponding to eye blinks.

Since finding *W* is a difficult inverse problem, iterative algorithms are used for finding *W* such that a particular cost such as kurtosis, negentropy, mutual information, or log likelihood is extremized (Comon, 1994; Bell and Sejnowski, 1995; Girolami and Fyfe, 1996; Touretzky et al., 1996; Hyvärinen and Oja, 1997). Unwanted sources can be identified by visual inspection or automatically (Delorme et al., 2001; Joyce et al., 2004; Bian et al., 2006; Li et al., 2006; Zhou and Gotman, 2009) and denoised EEG can be reconstructed. The performance of various ICA algorithms in removing artifacts from EEG are compared in Frølich and Dowding (2018) and several BSS algorithm including 20 ICA algorithms are given in Delorme et al. (2007). Methodological considerations in using ICA can be found in Klug and Gramann (2020). High-pass filtering with a cut-off frequency in the range of 1–2 Hz is an important preprocessing step in using ICA (Winkler et al., 2015).

Brigham and Kumar (2010) employed both heuristics and ICA for removing artifacts. EEG electrodes near eyes, temple and neck were removed since they were more prone to artifacts. Also, all epochs having the absolute values of signal components above 30 μ*V* were removed since they are mostly due to EMG artifacts. After this, ICA was applied on the preprocessed signal to obtain the independent components. Hurst exponent (Vorobyov and Cichocki, 2002) was then used to identify unwanted components. Independent components having Hurst exponent values in the range of 0.56 − 0.69 were discarded.

Sereshkeh et al. (2017a,b) used ICA and ADJUST algorithm for removing artifacts. ADJUST (Automatic EEG artifact detection based on the joint use of spatial and temporal features) (Mognon et al., 2011) is a fully automatic algorithm based on spatial and temporal features for identifying and removing independent components with artifacts. The algorithm automatically tunes its parameters to the data for computing artifact-specific spatial and temporal features required for classifying the independent components.

Deng et al. (2010), Jahangiri et al. (2019, 2018), and Jahangiri and Sepulveda (2019) have used SOBI (second-order blind identification) for artifact removal. SOBI has the advantage of being one of the fastest ICA algorithms (Sahonero and Calderon, 2017), although it may still be difficult to use it for real-time applications.

Nguyen et al. (2017) used an adaptive filtering based algorithm for removing artifacts (He et al., 2004). Unlike the ICA-based approaches, the adaptive filtering based approach can be used for real-time processing of multichannel EEG signal, due to its lower computational cost.

### 3.5. Selection of a Subset of Channels for Analysis

As described in section 2.1, the number of EEG channels acquired varies among the different works published in the literature. There are studies that make use of only 15 channels and there are others that use as high as 128 EEG channels. Similar to downsampling the acquired EEG signal in time domain prior to processing, a few researchers have also downsampled the signal in spatial domain; that is, only a subset of the acquired EEG channels are used for further processing. This section discusses the various approaches in selecting a subset of EEG channels.

García et al. (2012) manually selected only four out of the 14 EEG channels, which were F7, FC5, T7 and P7, based on their proximity to Geschwind-Wernicke's model areas (Geschwind, 1972). It may be noted that researchers have shown that Geschwind-Wernicke's model is not an accurate representation of language processing in human brain (Pillay et al., 2014; Binder, 2015; Tremblay and Dick, 2016), as already mentioned in section 2.1.

In the work by Myers (2004), 64-channel EEG was recorded but from these 64-channels, only channels involved in Broca's, Wernicke's, and sensorimotor areas (i.e., FC3, F5, CP3, P5, C3, and C4) were used for optimal time range and frequency band of the EEG signal that should be used for feature extraction and classification.

Similar to García et al. (2012), EEG channels are manually chosen in Panachakel et al. (2019). Specifically, the following 11 EEG channels are chosen based on the significance of the cortical region they cover in language processing (Marslen-Wilson and Tyler, 2007; Alderson-Day et al., 2015):

1. “C4”: postcentral gyrus
2. “FC3”: premotor cortex
3. “FC1”: premotor cortex
4. “F5”: inferior frontal gyrus, Broca's area
5. “C3”: postcentral gyrus
6. “F7”: Broca's area
7. “FT7”: inferior temporal gyrus
8. “CZ”: postcentral gyrus
9. “P3”: superior parietal lobule
10. “T7”: middle temporal gyrus, secondary auditory cortex
11. “C5”: Wernicke's area, primary auditory cortex

This choice of channels was also supported by the common spatial patterns (CSP) analysis on the imagined speech vs. rest state EEG data given in Nguyen et al. (2017). CSP is a linear transformation that maximizes the variance of the EEG signals from one class while minimizing the variance of the signals from another class (Sharon et al., 2019). Mathematically, CSP extremizes the following objective function:

(4) $$\begin{array}{l} J\left(w\right)=\frac{w^{T}X_{1}X_{1}^{T}w}{w^{T}X_{2}X_{2}^{T}w}=\frac{w^{T}C_{1}w}{w^{T}C_{2}w} \end{array}$$

where *T* denotes matrix transpose, matrix *X*_*i*_ contains the EEG signals of class *i*, with data samples as columns and channels as rows, *w* is the spatial filter and *C*_*i*_ is the spatial covariance matrix of class *i*. The EEG signals are usually band-pass filtered into a frequency band of interest whose variance between classes is extremized by the spatial patterns. The spatial filters can be seen as EEG source distribution vector (Wang et al., 2006). The channels corresponding to higher coefficients in the spatial filters may be the channels more correlated with the sources corresponding to the classes (Wang et al., 2006).

In Panachakel et al. (2020a), CSP was employed for determining the number of EEG channels to be considered. Nine EEG channels corresponding to the largest coefficients in *w* were chosen for feature extraction. It is also shown in Panachakel et al. (2020a) that nine was the optimal number of channels for the specific machine learning model presented in the paper since considering more or less than nine channels deteriorated the performance of the system. This approach has the advantage of adaptively learning the optimal channels to be considered which may change across different recording sessions based on the placement of EEG electrodes and different participants.

## 4. Feature Extraction and Classification

Most of the initial works on decoding imagined speech from EEG relied on features separately extracted from individual channels rather than simultaneously extracting the features from multichannel EEG data. Simultaneously extracting features from multichannel EEG helps in capturing the information transfer between multiple cortical regions and is resilient to slight changes in the placement of EEG electrodes across multiple subjects or across multiple recording sessions. Both statistical and wavelet domain features are popularly used for decoding imagined speech from EEG.

### 4.1. Feature Extraction From Individual Channels

Statistical features such as mean, median, variance, skewness, and kurtosis and their first and second derivatives were extracted in Zhao and Rudzicz (2015). This resulted in a feature vector of dimension 1, 197 per channel, which were initially concatenated together. Since there were 55 channels excluding the reference and EOG channels, this resulted in a feature vector of dimension 65, 835. To reduce the dimension of the feature vector, feature selection was performed based on the Pearson correlations with the given classes for each task independently. This resulted in a feature vector of dimension less than 100. The authors tried support vector machines (SVMs) with either radial basis function (RBF) or quadratic kernel and deep belief networks (DBNs) and SVM with RBF kernel gave better performance.

Min et al. (2016) used a subset of the features used in Zhao and Rudzicz (2015). Specifically, a trial was divided into 30 windows and for each window, mean, variance, standard deviation, and skewness were calculated. To reduce the dimension of the feature vector, sparse regression model based on Lasso was used for feature selection (Tibshirani, 1996) and ELM (extreme learning machine), ELM-L (extreme learning machine with linear function), ELM-R (extreme learning machine with radial basis function), SVM-R (support vector machine with radial basis function), and LDA (linear discriminant analysis) were used for classification. In the study, ELMs performed better than SVM and LDA.

García et al. (2012), Sereshkeh et al. (2017a), Jahangiri et al. (2019, 2018), Jahangiri and Sepulveda (2019), Pawar and Dhage (2020), and Panachakel et al. (2020a) used wavelet transform for extracting features. Specifically, Garcia et al. used Daubechies 2 (db2) wavelets, Jahangiri et al. have used Gabor wavelets and Sereshkeh et al., Pawar and Dhage and Panachakel et al used db4 wavelets as the mother wavelets. Use of wavelet transform is supported by its ability to localize information in both frequency and time domains (Subasi, 2005). Garcia et al. performed six levels of wavelet decomposition and used detail coefficients D2-D6 and approximation coefficient A6 as the features. The choice of the coefficients was based on the sampling rate (256 Hz) and the frequency of interest (4 to 25 Hz). Sereshkeh et al. performed 4 levels of wavelet decomposition using db4 wavelets. Instead of using the coefficients as such, as in the case of García et al. (2012), the standard deviation and root mean square of the approximation coefficients were used as features. Similar to Sereshkeh et al., Panachakel et al. also used 4 levels of wavelet decomposition using db4 wavelets but used root-mean-square (RMS), variance, and entropy of each approximation coefficient as features. Garcia et al. used SVM, random forest (RF), and naïve Bayes (NB) as the classifiers whereas Sereshkeh et al. used regularized neural networks. Garcia et al. reported higher accuracy with RF as the classifier. Panachakel et al. used a deep neural network with three hidden layers as the classifier.

In another work by Panachakel et al. (2019), a combination of time and wavelet domain features was employed. Corresponding to each trial, EEG signal of 3-s duration was decomposed into 7 levels using db4 wavelet and five statistical features, namely, root mean square, variance, kurtosis, skewness, and fifth order moment were extracted from the last three detail coefficients and from the last approximation coefficient. The same five statistical features were extracted from the 3-s time domain EEG signal and these features were concatenated with the features extracted from the wavelet coefficients to obtain the final feature vector. Similar to Panachakel et al. (2020a), a deep neural network with two hidden layers was used as the classifier.

Similar to Keirn and Aunon (1990) and Brigham and Kumar (2010) have used the coefficients of a sixth order autoregressive (AR) model as the features with 3-nearest neighbor classifier. The model coefficients were computed using the Burg method (Mac Kay, 1987). Order six was chosen since they observed that AR model of order six gave the best classification accuracy in their experiments.

In Cooney et al. (2018), experimented with three sets of features; the first set consisted of statistical measures such as mean, median, and standard deviation; the second set consisted of measures such as Hurst exponent and fractal dimension computed using (Psorakis et al., 2010); and the third set consisted of 13 Mel-frequency cepstral coefficients (MFCCs), a feature widely used in the domain of speech processing (Muda et al., 2010). PCA was used to reduce the dimension of the feature vector. SVM and decision tree were used as classifiers. The best accuracy is reported with MFCC as the feature vector and SVM as the classifier.

Though Hilbert–Huang transformation (HHT) (Huang et al., 1998; Huang, 2014) is a popular tool for feature extraction in classifying emotion from EEG (Uzun et al., 2012; Vanitha and Krishnan, 2017; Phadikar et al., 2019; Chen et al., 2020), the only work that makes use of HHT for classifying imagined speech is the work by Deng et al. (2010). Hilbert spectrum was extracted from the four primary SOBI (second-order blind identification) components and multiclass linear discriminant analysis (LDA) was used as the classifier.

Koizumi et al. (2018) extracted band powers from each channel. Band powers of 12 uniform frequency bands of 10 Hz from 0 to 120 Hz were computed from power spectral density (PSD) estimated using Welch periodogram method (Welch, 1967). Powers of all the bands were added to result in a feature vector whose each element corresponded to a specific EEG channel. SVM with quadratic polynomial kernel function was used for classification. In the work by Myers (2004), CSP was used as the feature extraction tool and autoregressive SVM was used as the classifier.

In Chengaiyan et al. (2020), brain connectivity features such as coherence (Thatcher et al., 2004), partial directed coherence (PDC) (Sameshima and Baccalá, 1999), direct transfer function (DTF) (Kaminski and Blinowska, 1991), and transfer entropy (Schreiber, 2000) were computed for each band of the EEG signal. The EEG frequency bands considered were delta, theta, alpha, beta and gamma. Two separate classifiers were built, one using recurrent neural networks (RNN) and the other, deep belief network (DBN). They reported a higher classification accuracy with DBN than with RNN.

### 4.2. Simultaneous Feature Extraction From Multiple Channels

#### 4.2.1. Using Channel Cross-Covariance (CCV) Matrices

In Nguyen et al. (2017), two distinct sets of features were employed, based on the tangent vectors of channel cross-covariance (CCV) matrices in Riemannian manifold. Using CCV matrix is preferred over the raw EEG signal because CCV matrices better capture the statistical relationship between the channels. Use of Riemannian manifold is motivated by the fact that since covariance matrix is symmetric positive definite (SPD), it lies in Riemannian manifold (Nguyen and Artemiadis, 2018). For a matrix in Riemannian manifold, the Euclidean distance is not an accurate descriptor; rather, the distance between the tangent vectors is. Also, tangent vectors are computationally more efficient than other metrics such as KL divergence (Nguyen and Artemiadis, 2018). Two approaches are presented in the paper for obtaining the covariance matrix; the first approach makes use of the raw EEG signal in the temporal domain whereas the second approach makes use of both the raw EEG and the wavelet coefficients of each channel extracted using the Morlet wavelet. Multi class RVM (mRVM) (Damoulas and Girolami, 2008; Psorakis et al., 2010) was used as the classifier. mRVMs are preferred over other conventional classifiers such as SVMs because mRVMs are inherently multiclass whereas SVMs are binary classifiers which are extended for multiclass using approaches like one-vs-all. Also, mRVMs can give the probability value of the prediction to be correct whereas raw SVMs can give only the predictions based on the position of the test vector with reference to the hyperplane. Nyugen et al. have reported higher accuracy when temporal and wavelet domain features are combined for the classification task.

In Saha and Fels (2019), Saha et al. (2019b), have used CCV matrices as the representation of the neural activity during speech imagery, similar to Nyugen's approach in Nguyen et al. (2017). In both works, the deep networks consist of different levels which are trained hierarchically. In Saha and Fels (2019), the first level consists of six-layered 1D-convolutional networks stacking two convolutional and two fully connected hidden layers and a six-layered recurrent neural network. The output of the 5th layer of the two previous networks are concatenated and fed to two deep autoencoders (DAE) and the latent vector representation of DAE is fed to a fully connected network for final classification. In Saha et al. (2019b), instead of the 1D-convolutional networks in layer 1, a four-layered 2D CNN stacking two convolutional and two fully connected hidden layers is used and instead of the fully connected network in the last layer, extreme gradient boosting (XGBoost) (Chen et al., 2015) is used for the final classification.

#### 4.2.2. Without Using Channel Cross-Covariance (CCV) Matrices

In a very recent work by Cooney et al. (2020), imagined speech is classified using three different CNN architectures that take the temporal domain EEG signals as the input. The aim of the work was to study the influence of hyperparameter optimization in decoding imagined speech. The three CNN architectures used are: (1) shallow ConvNet (Schirrmeister et al., 2017), (2) deep ConvNet (Schirrmeister et al., 2017), and (3) EEGNet (Lawhern et al., 2018). The hyperparameters considered in the study are activation function, learning rate, number of training epochs, and the loss function. Four each of activation functions, namely squaring non-linearity (Schirrmeister et al., 2017), exponential linear units (ELU) (Clevert et al., 2015), rectified linear unit (ReLU) (Agarap, 2018), and leaky ReLU (Maas et al., 2013), learning rate (0.001, 0.01, 0.1, and 1.0), number of training epochs (20, 40, 60, and 80) and two loss functions, namely negative log-likelihood (NLL) and cross-entropy (CE) were evaluated. They reported that leaky ReLU resulted in the best accuracy among all the four activation functions compared in the case of ConvNet whereas ELU performed better in the case of EEGNet. Also, smaller learning rates (0.001–0.1) were ideal for ConvNet whereas EEGNet performed best with a learning rate of 1. With respect to the number of training epochs, 20 epochs were sufficient for EEGNet whereas higher number of epochs were necessary for ConvNet. Both NLL and CE performed equally well and there was no statistically significant difference in the performance of the network between the two loss functions.

### 4.3. Transfer Learning Approaches

Transfer learning (TL) is used in García-Salinas et al. (2019) and Cooney et al. (2019) for improving the performance of the classifier. TL is a machine learning approach in which the performance of a classifier in the target domain is improved by incorporating the knowledge learnt from a different domain (Pan and Yang, 2009; He and Wu, 2017; García-Salinas et al., 2019). Specifically in García-Salinas et al. (2019), feature representation transfer is used for representing a new imagined word using the codewords learnt using a set of four other imagined words. The codewords were generated using k-means clustering similar to the approach discussed in Plinge et al. (2014) and Lazebnik and Raginsky (2008). These codewords were represented using histograms and a Naive Bayes classifier was used for classification. The accuracy of the classifier trained using all the five imagined words was comparable to the accuracy obtained by applying TL. This method is essentially an intra-subject transfer learning in which the knowledge is transferred for classifying a word which was not in the initial set of prompts. In Cooney et al. (2019), two TL paradigms are proposed which aim at inter-subject transfer learning. Specifically, TL is applied for improving the performance of the classifier on a new subject (target subject) using the knowledge learnt from a set of different subjects (source subjects). Similar to García-Salinas et al. (2019), the two TL paradigms come under the class of multi-task transfer learning (Evgeniou and Pontil, 2004). A deep CNN architecture, similar to the one proposed in Schirrmeister et al. (2017), is used in this work. Initially, the network is trained using the data from a selected set of subjects. These subjects are chosen based on the Pearson correlation coefficient of the subject's data with the target subject's data. This training is common for both the TL paradigms. In the first TL paradigm, a part of the target's data is used for fine-tuning the first two layers of the network which correspond to the input temporal and spatial convolution layers. In the second TL paradigm, the two layers prior to the output layer are fine-tuned using the data from the target subject. An improvement in accuracy over the non-TL approach is reported for both the TL paradigms.

### 4.4. Comparison of Performance of Different Approaches

**Figure 9:**
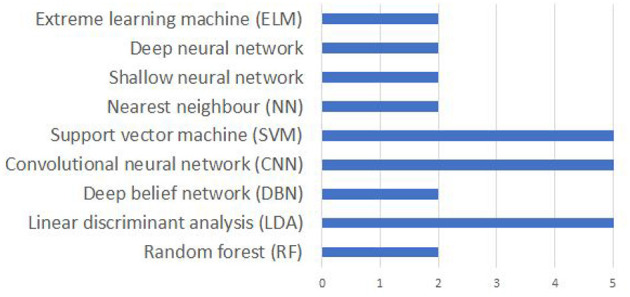
Comparison of popular machine learning algorithms used for decoding imagined speech from EEG. The x-axis gives the number of articles using each algorithm.

It is difficult to compare the accuracies reported in different papers due the differences in the data acquisition protocol including the differences in the number of EEG channels, number and nature of imagined speech prompts. Even for the works using the same dataset, a true comparison is impossible since the evaluation strategy (number of folds in cross-validation, classification of individual subjects vs. pooling the data from the entire set of subjects for classification, using a subset of the available prompts in the dataset) varies across these studies. Nevertheless, a comparison of the accuracies reported in several works reviewed in this manuscript are given in Table 4. Also, works that deal with classifying phonological categories, rather than actual imagined prompts are included in the tabular column. Figure 9 shows the frequency of use of various machine learning techniques for decoding imagined speech. Only around 32% of the works reviewed in this work make use of deep learning techniques whereas the remaining make use of traditional machine learning techniques.

**Table 4:** Comparison of the accuracies reported in several works (reviewed in this manuscript) on decoding imagined speech from EEG.

**Sl. No**.		**Prompts**	**Best features (if applicable)**	**Best classifier (if applicable)**	**Accuracy reported (%)**	**Remarks**
1	García et al. (2012)	“arriba”, “abajo”, “izquierda”, “derecha”, “seleccionar”	Discrete wavelet transform	RF	43.6 ± 2.4%	-
2	Brigham and Kumar (2010)	“/ba/”, “/ku/”	Autoregressive model coefficients	NN	68.8 ± 14.4%	-
3	Min et al. (2016)	“/a/”, “/e/”, “/i/”, “/o/”, “/u/”	Mean, variance, standard deviation, and skewness	ELM-R	87.0 ± 11.4%	Pairwise classification of all the five prompts and rest of subject S2
4	Sereshkeh et al. (2017a)	“yes”, “no”	Discrete wavelet transform	RNN	75.7 ± 9.6%	Classification of imagined speech v/s rest
5	Nguyen et al. (2017)	“/a/”, “/i/”, “/u/”; “in”, “out”, “up”; “independent”, “cooperate”	Tangent vectors in Riemannian manifold	mRVM	80.0 ± 7.3%	Classification of words "in" and "cooperate"
6	Panachakel et al. (2020a)	“in", “cooperate"	Temporal and Discrete wavelet transform	DNN	72.0 ± 8.5%	Classification of words "in" and "cooperate"
7	Panachakel et al. (2019)	“/iy/”, “/ uw/”, “/ piy/”, “/tiy/”, “/diy/”, “/m/”, “/n/”; “pat”, “pot”, “knew”, and “gnaw”	Discrete wavelet transform	DNN	57.1 ± 15.2%	-
8	Cooney et al. (2018)	“/iy/”, “/ uw/”, “/ piy/”, “/tiy/”, “/diy/”, “/m/”, “/n/”; “pat”, “pot”, “knew”, and “gnaw”	MFCC, statistical features etc.	SVM	22.7 ± 5.2%	-
9	Saha and Fels (2019)	“/a/”, “/i/”, “/u/”; “in”, “out”, “up”; “independent”, “cooperate”	Channel cross-covariance (CCV)	CNN+RNN+DAE	79.9 ± 6.9%	Classification of words "independent" and "cooperate"
10	García-Salinas et al. (2019)	“arriba”, “abajo”, “izquierda”, “derecha”, “seleccionar”	Bag of Features and trasnfer learning	Naive Bayes	61.4 ± 12.4%	Representation of “abajo” learnt using transfer learning
11	Cooney et al. (2019)	“/a/”, “/e/”, “/i/”, “/o/,” “/u/ ”		CNN	35.7 ± 3.0%	Uses transfer learning
12	Tøttrup et al. (2019)	“go”, “stop” and “Viborg”	Spectral and temporal features	RF	67.0 ± 9.0%	-
13	Balaji et al. (2017)	“Haan”, “Na” and “Yes” and “No”	Spectral power	ANN	73.4%	Subject-wise accuracy is not reported
14	Jahangiri et al. (2019)	“/ba/”, “/fo/”, “/le/” and ‘/‘ry/”	Discrete Gabor transform	LDA	82.5 ± 4.1%	
15	Pawar and Dhage (2020)	“left”, “right”, “up” and “down”	Discrete wavelet transform	ELM-G	47.9 ± 6.9%	
16	Jahangiri et al. (2018)	“/ba/”, “/fo/”, “/le/” and ‘/‘ry/”	Discrete Gabor transform	LDA	82.5 ± 24.1%	
17	Saha et al. (2019b)	“/iy/”, “/ uw/”, “/ piy/”, “/tiy/”, “/diy/”, “/m/”, “/n/”; “pat”, “pot”, “knew”, and “gnaw”	Channel cross-covariance (CCV)	CNN+ LSTM	77.5 ± 4.2%	Classification of phonological categories
18	Koizumi et al. (2018)	“ue”, “shita”, “hidari”, “migi”, “mae”, “ushiro”	Spectral power	SVM	81.3%	Subject-wise accuracy is not reported
19	Deng et al. (2010)	Constructed using “/ba/” and “/ku”	Hilbert spectrum	LDA	58.1 ± 8.0%	Classification of rhythm
20	Zhang et al. (2020)	Mandarin lexical tones	Common spatial patterns	SVM	80.1 ± 1.2%	
21	Watanabe et al. (2020)	Constructed using “/ba/”	-	NN	38.5 ± 5.3%	
22	Jahangiri and Sepulveda (2017)	“/ba/”, “/fo/”, “/le/” and ‘/‘ry/”	Discrete Gabor transform	LDA	80.7 ± 3.1%	Pairwise classification
23	Jahangiri and Sepulveda (2019)	“/ba/”, “/fo/”, “/le/” and ‘/‘ry/”	Discrete Gabor transform	LDA	96.4 ± 2.3%	One v/s all classification
24	Chengaiyan et al. (2020)	50 CVC words	Brain connectivity estimators and entropy measures	DBN	80.0%	Subject-wise accuracy is not tabulated
25	Saha et al. (2019a)	“/iy/”, “/ uw/”, “/ piy/”, “/tiy/”, “/diy/”, “/m/”, “/n/”; “pat”, “pot”, “knew”, and “gnaw”	Channel cross-covariance (CCV)	CNN+DAE+XG Boost	53.36%	Subject-wise accuracy is not reported
26	Zhao and Rudzicz (2015)	“/iy/”, “/ uw/”, “/ piy/”, “/tiy/”, “/diy/”, “/m/”, “/n/”; “pat”, “pot”, “knew”, and “gnaw”	Statistical features	SVM	55.4 ± 20%	Classification of phonological categories
27	Cooney et al. (2020)	“/a/”, “/e/”, “/i/”, “/o/,” “/u/ ”; “arriba”, “abajo”, “derecha”, “izquierda”, “adelante”, “atrás”	-	CNN	30.1 ± 2.7%	Classification of imagined vowel
28	Sereshkeh et al. (2017b)	“yes”, “no”	AR coefficients and DWT	SVM	75.9 ± 11.4%	Online classification

Below, we analyze the performance of the systems based on the types of prompts used, namely:

1. Directional prompts
2. Polar prompts
3. Vowel prompts

Since the number of classes under these prompts are different, we used Cohen's kappa (κ) value as the metric for comparing the systems. Cohen's kappa value is defined as:

(5) $$\begin{array}{l} \kappa :=\frac{p_{cl}-p_{ch}}{100-p_{ch}} \end{array}$$

where *p*_*cl*_ and *p*_*ch*_ are the system and chance level accuracies, respectively, both in percentage.

The value of κ theoretically lies in the range [−1, 1]. Values closer to −1 indicate that the system is performing badly, whereas a value closer to 1 indicates that the system is very good. A value of 0 indicates that the classifier is only as good as random guess whereas a value less than 0 indicates that the performance of the classifier is inferior to random guess.

#### 4.4.1. Directional Prompts

Directional prompts include words that can be used for controlling devices such as wheelchairs and user interfaces like computer pointing devices. Five studies reviewed in this article makes use of directional prompts. In both García et al. (2012), García-Salinas et al. (2019), five Spanish words, “arriba”, “abajo”, “izquierda”, “derecha”, and “seleccionar” are used as the prompts. These words mean up, down, left,‘right and select, respectively. The prompts used in Pawar and Dhage (2020) are “left”, “right”, “up”, and “down”. In Koizumi et al. (2018), six Japanese words “ue”, “shita”, “hidari”, “migi”, and “mae” are used as the prompts. They mean up, down, left, right, forward and backward, respectively. Similar to García et al. (2012) and García-Salinas et al. (2019), Cooney et al. (2020) have also used Spanish words. The six Spanish words used by Cooney et al. (2020) are “arriba”, “abajo”, “derecha”, “izquierda”, “adelante”, and “atrás” which mean up, down, left, right, backward, and forward. García et al. (2012), García-Salinas et al. (2019) made use of the same dataset acquired using -channel Emotiv EPOC commercial grade EEG acquisition system sampled at 128 Hz. The EEG data for Pawar and Dhage (2020) is acquired using 64-channel Neuroscan synamps 2 research grade EEG acquisition system sampled at 1,000 Hz. Koizumi et al. (2018) used a 65-channel EEG-1200, Nihon Kohden Corporation research grade EEG acquisition system sampled at 1,000 Hz whereas Cooney et al. (2020) used the dataset acquired using 18-channel Grass 8-18-36 commercial grade EEG acquisition system sampled at 1,024 Hz. The κ values of these systems are given in Table 5 (sl. no. 1 - 5). Clearly, Koizumi et al. (2018) has the best performance in terms of κ value and Cooney et al. (2020) has the worst performance. This cannot be attributed to the system type (commercial grade/research grade) because García-Salinas et al. (2019), who also made use of a commercial grade system like Cooney et al. (2020), have obtained much better performance than Cooney et al. (2020). Also, the data sampling rate may not have affected the accuracy. One key difference between Koizumi et al. (2018) and other works is the use of gamma band. Since both Pawar and Dhage (2020) and Koizumi et al. (2018) have used the gamma band, the higher performance of Koizumi et al. (2018) cannot be attributed to the use of gamma band alone.

**Table 5:** Comparison of κ values of different works using (a) directional prompts (shaded in gray), (b) polar prompts (shaded in pink) and (c) vowel prompts (shaded in cyan).

**Sl. No**.		**Classes**	**Classifier**	**Accuracy Achieved (%)**	**Chance Accuracy (%)**	**k Value**	**Remarks**
1	García et al. (2012)	“arriba”, “abajo”, “izquierda”, “derecha”, “seleccionar”	RF	43.6	20	0.3	-
2	García-Salinas et al. (2019)	“arriba”, “abajo”, “izquierda”, “derecha”, “seleccionar”	Naive Bayes	61.4	20	0.5	-
3	Pawar and Dhage (2020)	“left”, “right”, “up” and “down”	ELM-G	47.9	25	0.3	Uses gamma band
4	Koizumi et al. (2018)	“ue”, “shita”, “hidari”, “migi”, “mae”, “ushiro”	SVM	81.3	16.7	0.8	Uses gamma band
5	Cooney et al. (2020)	“arriba”, “abajo”, “derecha”, “izquierda”, “adelante”, “atrás'	CNN	25	16.7	0.1	-
6	Sereshkeh et al. (2017a)	Decision “yes” vs. “no”	RNN	63.2	57.8	0.1	-
7	Balaji et al. (2017)	Decision “yes” vs. “no”	ANN	85.2	50	0.7	Uses bilingual prompts
8	Sereshkeh et al. (2017b)	Decision “yes” vs. “no”	SVM	69.3	60	0.2	Employs online decoding
9	Min et al. (2016)	Pairwise combinations of /a/, /e/, /i/, /o/, /u/ and mute	ELM-R	68.5	50	0.4	Accuracy is the mean of all the pairwise classification accuracies across all the subjects
10	Nguyen et al. (2017)	/a/, /i/ and /u/	mRVM	49.0	33.3	0.2	-
11	Saha and Fels (2019)	/a/, /i/ and /u/	CNN+RcNN+DAE	74.3	33.3	0.6	-
12	Cooney et al. (2020)	/a/, /e/, /i/, /o/, and /u/	CNN	30.3	20	0.1	-

*RF, Random forest; ELM-G, Extreme learning machine (Gaussian kernel); SVM, Support vector machine; CNN, Convolutional neural networks; RNN, Regularized neural network; ANN, Artificial neural network; ELM-R, Extreme learning machine (radial basis function); mRVM, multiclass relevance vector machine; RcNN, Recurrent neural network; DAE, Deep autoencoder*.

#### 4.4.2. Polar Prompts

Polar prompts are the responses to binary questions or polar questions. Three studies reviewed in this article have made use of answers to binary questions as the prompts. As described in section 2.4.2, the participants were cued using binary questions. Both Sereshkeh et al. (2017a) and Sereshkeh et al. (2017b) used a 64-channel BrainAmp research grade EEG acquisition system with a sampling rate of 1 KHz for acquiring the EEG data. On the other hand, Balaji et al. (2017) used a 32-channel research grade (Electrical Geodesics, Inc.). EEG acquisition system with a sampling rate of 250 Hz. Unlike Sereshkeh et al. (2017a) and Sereshkeh et al. (2017b), in Balaji et al. (2017) the binary questions were posed in two languages, namely Hindi and English. Also, Sereshkeh et al. (2017b) is the only work that uses an online strategy for decoding imagined speech from EEG.

The following conclusions can be made from the results presented in Balaji et al. (2017):

- Though all the participants were native Hindi speakers who learned English only as their second language, the classification accuracy is better when the binary questions are posed in English rather than in Hindi. This is contrary to what one might expect.
- When the responses to all the questions (both Hindi and English) are pooled together and used for classification, only rarely does the classifier make a cross-language prediction error. This might be because of the distinct language-specific sites present in the brain of bilinguals (Lucas et al., 2004).

Based on Cohen's κ values given in Table 5 (sl. no. 6 - 8), the system proposed by Balaji et al. (2017) performs better than those proposed by Sereshkeh et al. (2017a) and Sereshkeh et al. (2017b). This cannot be considered as the consequence of the classifier used since the classifiers used by Sereshkeh et al. (2017a) and Balaji et al. (2017) are very similar.

Further studies are required to explain these counter-intuitive observations, much in the line of various studies reported in the literature on the neural differences between bilinguals and monolinguals (Marian and Shook, 2012; Hammer, 2017; Gangopadhyay et al., 2018).

#### 4.4.3. Vowel Prompts

Four studies reviewed in this study have used vowel imagery in their paradigm. Min et al. (2016) and Cooney et al. (2020) have used the entire set of vowels as their prompts whereas Nguyen et al. (2017) and Saha and Fels (2019) have used only three vowels: /a/, /i/, and /u/. Min et al. (2016) have used a 64-channel, research grade Electrical Geodesics, Inc. EEG acquisition system whereas Nguyen et al. (2017) have used a 64-channel, research grade BrainProducts ActiCHamp EEG acquisition system, both sampled at 1000 Hz. Both Min et al. (2016) and Nguyen et al. (2017) have downsampled the acquired data, to 250 Hz and 256 Hz, respectively. Saha and Fels (2019) have used the EEG dataset created by Nguyen et al. (2017). On the other hand, Cooney et al. (2020) have used an 18-channel, commercial grade EEG amplifier (Grass 8-18-36) for acquiring the data at 1024 Hz. This was later downsampled to 128 Hz.

Based on Cohen's κ values given in Table 5 (sl. no. 9 - 10), the system proposed by Saha and Fels (2019) performs better than those proposed by Min et al. (2016), Nguyen et al. (2017), and Cooney et al. (2020). Since Nguyen et al. (2017) and Saha and Fels (2019) have used the same EEG dataset, the improvement can be attributed to the superior classification technique used by Saha and Fels (2019). Nguyen et al. (2017), Saha and Fels (2019) and Cooney et al. (2020) have also tested their approach on the EEG data acquired when the participants were imagining articulating short words (Cooney et al. (2020): “arriba”, “abajo”, “derecha”, “izquierda”, “adelante”, and “atrás”; Nguyen et al. (2017) and Saha and Fels (2019): “in”, “out”, and “up”). For both Nguyen et al. (2017) and Saha and Fels (2019), there is a marginal improvement in the κ values when short words are used instead of vowels whereas for Cooney et al. (2020), there is a marginal reduction. Therefore, we cannot concretely claim any advantage for short words over vowels when used as prompts for imagined speech.

## 5. Considerations in Designing a Speech Imagery Based Online BCI System

A speech imagery based BCI system essentially comes under the category of an endogenous BCI system where speech imagery is used for generating the neural activation, although cues might be used for generating the speech imagery (Nguyen et al., 2017). Deploying an EEG based endogenous BCI system for practical applications is far more difficult that using an EEG based exogenous system due to the following reasons:

1. Evoked potentials and event-related potentials used in an exogenous system have higher signal-to-noise ratio.
2. More number of EEG channels are required in an endogenous BCI system than an exogenous BCI system. Considering the longer preparation time required in a wet EEG electrode system and the difficulties in cleaning the scalp area after EEG acquisition, the requirement of more number of channels leads to the use of dry electrodes. Although recent studies have shown comparable signal qualities in wet and dry electrodes (Lopez-Gordo et al., 2014; Hinrichs et al., 2020), EEG recorded using dry electrodes are more prone to artifacts (Leach et al., 2020).

In addition, there are more challenges when the system needs to be online, which are enumerated below:

1. Most of the systems reviewed in this article are synchronous BCI systems which provide a less natural mode of communication than an asynchronous BCI system. The EEG signal generated for a synchronous BCI is less corrupted by artifacts since the subject could avoid eye blinks, eye movements etc. during the period when the actual EEG to be analyzed is captured. In an asynchronous BCI system, the system needs to mitigate the effects of these artifacts leading to a more complex architecture of the system. Also, the effect of attention toward the prompts is not well-understood. That is, the observed neural activation might be because of the cues rather than due to the imagination. It is worth noting that the “no vs. rest” system proposed in Sereshkeh et al. (2017b) can be made to work in an asynchronous mode.
2. The upper bound on the computational complexity of the algorithms used in the system may limit the efficiency of the system in removing artifacts, extracting features with high discriminability etc. This makes the design of a system with high accuracy more difficult. For instance, many formulations of the popular tool for artifact removal has high computational cost and requires high amounts of data for convergence. This problem can be addressed by using algorithms that detect and remove artifacts in real-time such as ADJUST (Automatic EEG artifact detection based on the joint use of spatial and temporal features) (Mognon et al., 2011) used by Nguyen et al. (2017), or other algorithms like online recursive ICA algorithm (ORICA) (Hsu et al., 2015) and hybrid ICA-ANC (independent component analysis-adaptive noise cancellation) (Jafarifarmand et al., 2017).
3. In the case of a system with only two degrees of freedom, repeated imagination of the prompt may not lead to any undesirable BCI outputs but this is not the case for a system with higher number of degrees of freedom.

## 6. Conclusion and Future Directions

In spite of focused research spanning over a decade, we still do not have a system that can decode imagined speech from EEG with sufficient accuracy for a practical system. The algorithms that offer reasonable accuracy either have a very limited set of vocabulary or perform poorly for unseen subjects (whose data has not been seen by the system during its training phase). Based on the review of recent works in the literature, the following recommendations are made:

- **Type of EEG acquisition system:** Most of the works in the literature are based on the data acquired using EEG systems with 64 channels. Though there is an improvement in the accuracy when high-density EEG system is used, considering the practical difficulties in deploying a BCI system with high-density EEG system, it may not be feasible to have such a BCI for any practical purposes. Also, the efficiency of ICA algorithm plateaus near 64 channels and hence having more than 64 EEG channels may not help in artifact removal also. As noted in section 2, there is a trade-off between the accuracy of the system and the ease with which the system can be deployed. Also, as noted in section 3.1, most of the works downsample the acquired EEG data to 256 Hz and hence it is not required to have EEG acquisition systems of high sampling rates.
- **Preferred mode of stimulus delivery:** Though auditory cues have commonly been used in the literature, we recommend that it is best avoided since it is difficult to remove the signature of the auditory cue from the EEG signal recorded during speech imagery. We recommend the use of visual cues since the occipital lobe is not involved in speech production or comprehension and hence the neural signals elicited in the occipital lobe can easily be removed. Out of the 28 papers reviewed in this article, only one of the article deals with online decoding of imagined speech. Though many works use auditory cues, it needs to be investigated what exactly is giving rise to the neural response, whether it is the auditory cues or the imagination of the cued prompts. As mentioned in section 1.2, many regions in the auditory cortex are activated during speech imagery due to efference copies. A system trained on the distinct neural activities due to cues or the attention toward it may not be of any practical significance.
- **Repeated imagination of prompts:** It is observed that repeated imagination improves the discriminability of the neural signals elicited during speech imagery. Also, recordings with repeated imagination can be used to identify the set of EEG channels most informative for our purpose. Nevertheless, it is difficult to have a practical online BCI system that works on repeated imagination, especially when the number of degrees of freedom are high. Hence, although repeated imagination of prompts has benefits in a laboratory setting, it is difficult to extend these systems for practical application.
- **Choice of prompts:** It has been shown in the literature that a set of prompts with different lengths and complexity yields better classification accuracy. It has also been shown that bilingual prompts improve the classification performance. In an ideal situation, speech imagery has the possibility of having many prompts and hence many degrees of freedom. However, this aspect becomes relevant only when the systems achieve a level of performance adequate for deployment in a real life, online BCI system.
- **Preprocessing:** The most common preprocessing step in the literature is temporal filtering. Most of the researchers have band-pass filtered the EEG signal in the range 2 to 50 Hz. In addition, a notch filter is used by most of the researchers to remove the powerline hum. If ICA is used, a high pass filter with a cut-off frequency in the range 1 to 2 Hz is highly recommended. If gamma band is also included in feature extraction, algorithms for removing EMG artifacts should be used. As noted by Saha et al. (2019b), it is better to avoid spatial filtering in the preprocessing pipeline. Most of the popular ICA algorithms currently available are not suited for real-time applications and hence other algorithms like those used by Nguyen et al. (2017) should be used.
- **Features and classifiers used:** Most of the works that make use of traditional machine learning techniques such as ANN, ELM, and SVM extract features from each channel independently. In the case of works that use deep-learning techniques, features are usually extracted from channel cross-covariance (CCV) matrices. Use of CCV matrices is preferred since they better capture the information transfer between different brain regions. Although researchers in other fields such as speech recognition and computer vision have almost completely moved to deep-learning, researchers working on decoding imagined speech from EEG still make use of conventional machine learning techniques primarily due to the limitation in the amount of data available for training the classifiers.

The following research directions have been identified:

1. Identifying a better set of prompts which have highly discriminable EEG signatures. Identifying this set requires the efforts of neurobiologists and linguists. For example, one could experiment with a set of words, each of which contains phonemes as distinct from other words as possible, in terms of place and manner of articulation. Further, the effect of the language of the imagined prompt on the signatures of the EEG has not been explored much except in the work by Balaji et al. (2017). For instance, in the case of bilingual subjects, we could possibly use words from different languages and see if it improves the signal-to-noise ratio of the obtained responses. Also, more work needs to be carried out on employing prompts of different rhythms and tones. Although prompts have phonetic and/or lexical difference, prompts with varying length, bilingual prompts etc. have been used by several researchers, it is not well-understood which characteristic of the prompt is causing the distinct neural activities. Further studies are required to understand the effect of these differences.
2. Although EEG has very high temporal resolution compared to imaging techniques such as fMRI, EEG is highly corrupted by noise. Developing proper signal processing algorithms to improve the SNR of EEG recorded during speech imagery can help in improving the accuracy of systems for decoding imagined speech. The relative advantages of non-auditory cues also need to be clearly established.
3. There is high variability between the EEG signals acquired from different participants. Even in the case of EEG signal acquired from the same participant, there is high inter-trial variability (García-Salinas et al., 2019). Techniques to normalize the EEG acquired from different subjects and also from different trials of the same subject can help in reducing the calibration time of the system. This improves the ease with which the system can be deployed for practical applications. This is similar to the work by Sharon et al. (2019) where subject adaptation is used for improving the accuracy in motor imagery.
4. Identifying better features and better machine learning algorithms can help reduce the data requirement during the training and calibration phases. This will also result in better classification accuracy, improving the practical significance of the system. Also. algorithms used to classify motor imagery may not be suitable for speech imagery since the laterality present in motor imagery (for eg. left hand imagery and right hand imagery, which have contralateral brain activation) is not there in speech imagery. Thus, further research in the field of feature extraction techniques is necessary.
5. The effect of sampling rate and frequency band has not been studied yet in the case of speech imagery. Information on the ideal sampling rate and frequency band can help in designing better BCI systems.
6. Almost all of the current studies are based on healthy subjects. Further studies are required to understand how well these systems perform on patients with brain damage.

To help budding researchers to kick-start their research in decoding imagined speech from EEG, the details of the three most popular publicly available datasets having EEG acquired during imagined speech are listed in Table 6.

**Table 6:** Details of the three most popular publicly available speech imagery EEG datasets.

**Creators**	**Prompts**	**No. of EEG Channels**	**Sampling rate**	**No. of subjects**	**URL**
Shunan Zhao and Frank Rudzicz (Zhao and Rudzicz, 2015)	Phonemic/syllabic prompts (/iy/, / uw/, / piy/, /tiy/, /diy/, /m/, / n/) and words (pat, pot, knew, and gnaw)	64	1 KHz	14	http://www.cs.toronto.edu/~complingweb/data/karaOne/karaOne.html
German A. Pressel Corettoa, Ivan E. Gareisa, and H. Leonardo Rufiner (Coretto et al., 2017)	Vowels (/a/, /e/, /i/, /o/, /u/) and words (“arriba”, “abajo”, “derecha”, “izquierda”, “adelante”, “atras”)	6	1 KHz	15	http://fich.unl.edu.ar/sinc/downloads/imagined_speech
Chuong H Nguyen, George K Karavas and Panagiotis Artemiadis (Nguyen et al., 2017)	Vowels (/a/, /i/, /u/) and words (“in”, “out” and “up”, “cooperate”, “independent”)	64	1 KHz	15	https://www.dropbox.com/s/01k9c75j0x3jfb9/Dataset.zip?dl=0

## Author Contributions

JP drafted the initial version of the manuscript under the guidance of AR. AR wrote the final version of the manuscript. Both authors contributed to the article and approved the submitted version.

## Conflict of Interest

The authors declare that the research was conducted in the absence of any commercial or financial relationships that could be construed as a potential conflict of interest.
